# A tailored multi-functional catalyst for ultra-efficient styrene production under a cyclic redox scheme

**DOI:** 10.1038/s41467-021-21374-2

**Published:** 2021-02-26

**Authors:** Xing Zhu, Yunfei Gao, Xijun Wang, Vasudev Haribal, Junchen Liu, Luke M. Neal, Zhenghong Bao, Zili Wu, Hua Wang, Fanxing Li

**Affiliations:** 1 Department of Chemical and Biomolecular Engineering, North Carolina State University, Raleigh, NC USA; 2 State Key Laboratory of Complex Nonferrous Metal Resources Clean Utilization, Faculty of Metallurgical and Energy Engineering, Kunming University of Science and Technology, Kunming, China; 3 Oak Ridge National Laboratory, Chemical Science Division and Center for Nanophase Materials Sciences, Oak Ridge, TN USA

**Keywords:** Heterogeneous catalysis, Crude oil, Chemical engineering

## Abstract

Styrene is an important commodity chemical that is highly energy and CO_2_ intensive to produce. We report a redox oxidative dehydrogenation (redox-ODH) strategy to efficiently produce styrene. Facilitated by a multifunctional (Ca/Mn)_1−*x*_O@KFeO_2_ core-shell redox catalyst which acts as (i) a heterogeneous catalyst, (ii) an oxygen separation agent, and (iii) a selective hydrogen combustion material, redox-ODH auto-thermally converts ethylbenzene to styrene with up to 97% single-pass conversion and >94% selectivity. This represents a 72% yield increase compared to commercial dehydrogenation on a relative basis, leading to 82% energy savings and 79% CO_2_ emission reduction. The redox catalyst is composed of a catalytically active KFeO_2_ shell and a (Ca/Mn)_1−*x*_O core for reversible lattice oxygen storage and donation. The lattice oxygen donation from (Ca/Mn)_1−*x*_O sacrificially stabilizes Fe^3+^ in the shell to maintain high catalytic activity and coke resistance. From a practical standpoint, the redox catalyst exhibits excellent long-term performance under industrially compatible conditions.

## Abstract (web summary)

Styrene is an important commodity chemical that is highly energy and CO_2_ intensive to produce. Here, authors report a redox-oxidative dehydrogenation scheme and a tailored core-shell redox catalyst to convert ethylbenzene to styrene with up to 91.4% single-pass yield and 82% energy savings.

## Introduction

As an important chemical feedstock for rubber and plastics production, the global styrene production exceeded 31 million tons in 2018 and results in an annual CO_2_ emission of over 27 million tons^1,2^. At present, catalytic dehydrogenation (DH) of ethylbenzene accounts for ~90% of the styrene produced worldwide. This well-established approach suffers from high energy consumption, equilibrium-limited ethylbenzene conversion, and complex product separation, leading to significant CO_2_ emissions^3,4^. To compensate the heat required by the highly endothermic DH reaction, the industrial DH process uses significant amount of superheated steam as a heat source. At a typical dilution rate of ~22:1 (steam:ethylbenzene by mole), co-injection of steam increases the equilibrium conversion while inhibiting coke formation and over-reduction of the potassium-promoted iron oxide DH catalyst^5,6^. Despite the large steam consumption that is highly energy-intensive, commercial DH process needs two reactors in series with interstage heating in order to achieve 64% ethylbenzene conversion^7,8^. The limited ethylbenzene conversion, coupled with by-products such as benzene and toluene, in turn complicates the product separation scheme. As such, transformative approaches to convert ethylbenzene to styrene with lowered energy conversion, higher single-pass yield, and simpler process scheme is highly desirable.

Oxidative DH (ODH) is a promising alternative to DH, since simultaneous oxidation of hydrogen to water eliminates equilibrium limitations and leads to an exothermic overall reaction^3,9^. However, co-feeding gaseous oxygen in ODH often leads to undesirable carbon dioxide formation, lowering the selectivity to styrene. State-of-the-art ethylbenzene O_2_-ODH catalysts include high-surface-area activated carbon materials, carbon-doped boron nitride, and supported vanadium-based oxide. Styrene yields from these catalysts are typically limited to 60%, with ethylbenzene feed partial pressure <0.1 atm^9–12^. Moreover, the consumption of gaseous oxygen also increases the energy consumption, cost, and complexity of the process. Besides gaseous oxygen, carbon dioxide was also investigated as a soft oxidant for ethylbenzene ODH, typical mechanisms include a modified Mars-van Krevelen-type redox mechanism, and a reverse water-gas-shift reaction, which converts the H_2_ coproduct into water and CO^13,14^. Reported catalysts include CeO_2_^13^, activated carbon-supported FeO_*x*_^15^, and other mixed transition metal oxides^16–19^. Although CO_2_-ODH can facilitate CO_2_ utilization and enhance the equilibrium styrene yield, high carbon dioxide to ethylbenzene molar ratios (~10:1) are necessary given the equilibrium-limited reverse water-gas-shift reaction^16^. The limited CO_2_ conversion would in turn increase the complexity and energy demand for the product separation steps. Moreover, CO_2_-ODH is more endothermic than conventional DH. As such, CO_2_-ODH processes are likely to be more energy-intensive than the conventional DH route.

**Fig. 1:**
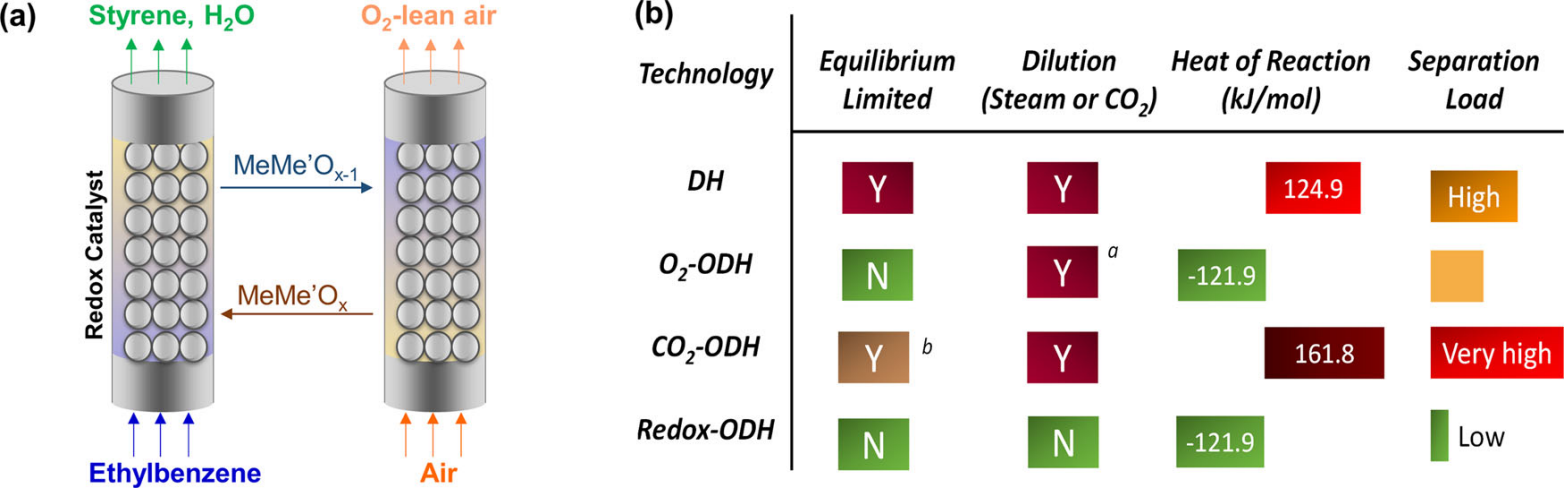
Schematic and the advantages of redox-ODH. **a** Schematic illustration of redox-ODH of ethylbenzene. MeMe′O_*x*_ represents a generic redox catalyst. **b** A comparison between ethylbenzene DH, O_2_-ODH, CO_2_-ODH, and redox-ODH. Green background stands for desirable features. ^a^Steam dilution is necessary to avoid combustion limit. ^b^ CO_2_-ODH is still equilibrium limited compared to O_2_- and redox-ODH due to the equilibrium limitation in R-WGS (reverse water-gas-shift).

To address these challenges, we propose a redox-ODH scheme to efficiently convert ethylbenzene to styrene using a tailored multifunctional redox catalyst, which acts as a heterogeneous catalyst, an oxygen separation agent, and a selective oxidation material (Fig. 1a). Facilitated by the redox catalyst, redox-ODH follows a chemical-looping-based approach, which has been previously reported for CO_2_ capture from fossil fuel combustion^20–22^, methane-selective oxidation^23–29^, thermochemical water/CO_2_ splitting^30–37^, air separation^38–40^, and ODH of light alkanes^41–43^. To our knowledge, previous chemical-looping studies have yet to investigate catalytic conversion to hydrocarbon molecules containing more than four carbon atoms, mainly due to the high operating temperature required by conventional redox catalysts and/or lack of product selectivity^44,45^. In comparison, redox-ODH converts ethylbenzene at relatively low temperatures in two steps, with integrated air separation: in the ODH step, ethylbenzene is catalytically converted into styrene and water, where the active lattice oxygen in the redox catalyst assists ethylbenzene activation and selective hydrogen combustion (SHC). In the subsequent oxidation step, the lattice oxygen in the redox catalyst is replenished via reactive air separation. Resulted from effective SHC by the redox catalyst, the conversion of ethylbenzene to styrene becomes autothermal and is no longer limited by the thermodynamic equilibrium, achieving high single-pass styrene yield while simplifying the downstream product separation. Moreover, the in situ air separation facilitated by the redox catalyst renders a much safer and more efficient ODH scheme^44^. Figure 1b compares redox-ODH with conventional and emerging styrene production approaches. As can be seen, significant advantages can be anticipated resulting from the multifunctional redox catalyst and the unique redox-ODH scheme. In the following sections, the performance of the multifunctional redox catalyst is first presented. This is followed with detailed characterization and density functional theory (DFT) calculations to reveal the underlying reaction mechanism and redox catalyst design and optimization strategy. Finally, the performance of the redox-ODH scheme is simulated with detailed ASPEN Plus simulations. Specifically, the redox catalyst reported in this study demonstrated 72% increase in single-pass styrene yield on a relative basis. The significantly intensified redox-ODH scheme has the potential to reduce energy consumption by >80% when compared to the commercial DH process.

## Results

### Redox-ODH performance of ethylbenzene to styrene

**Fig. 2:**
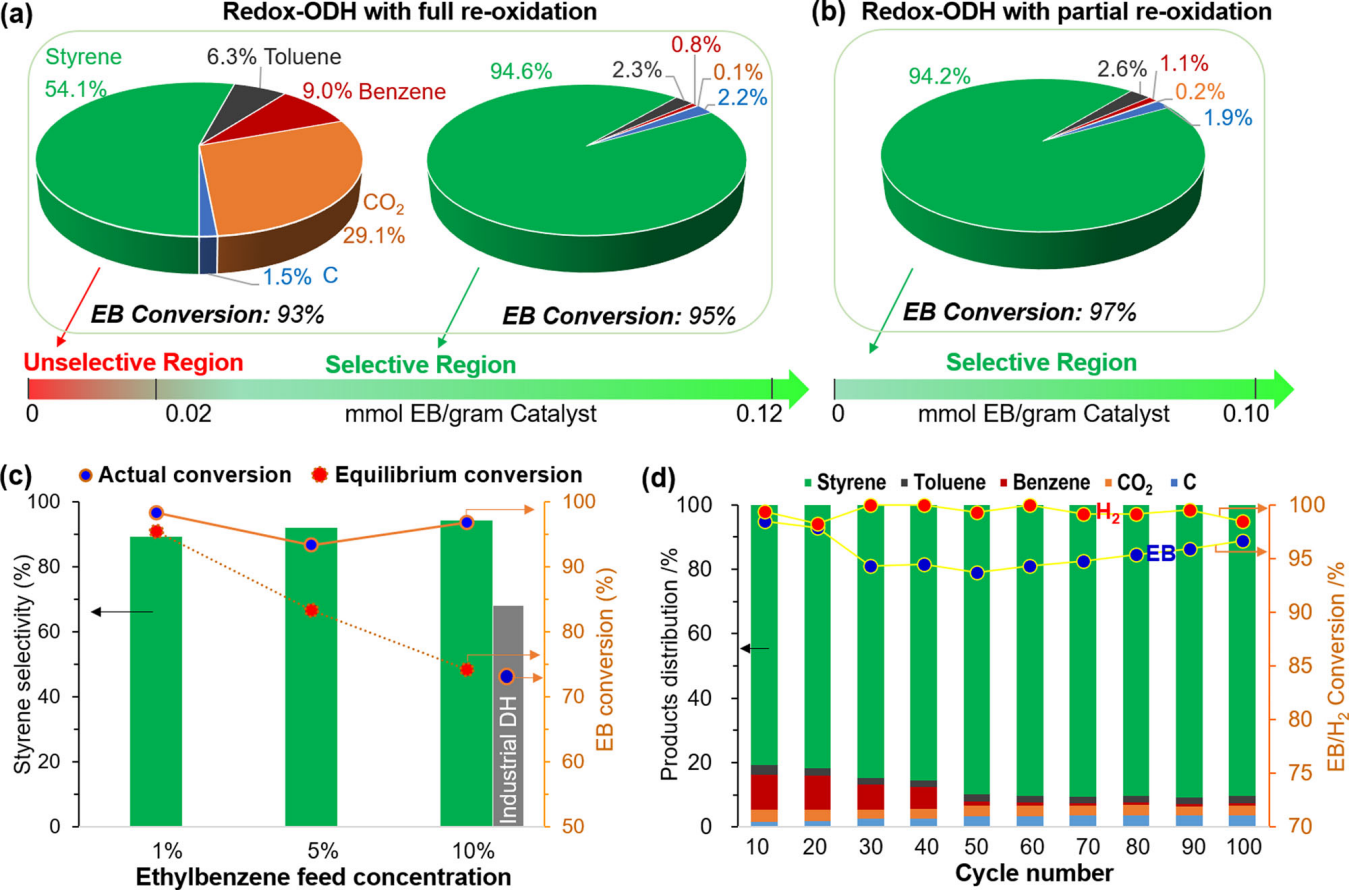
Redox-ODH performance. Performance comparison of redox-ODH of ethylbenzene (EB) with **a** fully reoxidized and **b** partially reoxidized redox catalyst, EB partial pressure = 0.01 atm, temperature = 600 °C. **c** ODH performance comparing to DH equilibrium conversions in the range of 0.01–0.1 atm ethylbenzene feed partial pressure (balance Ar) using partially reoxidized redox catalyst. **d** Long-term cycle and product distributions in redox-ODH using fully reoxidized redox catalyst.

This section reports the performance of the (Ca/Mn)_1 − *x*_O@KFeO_2_ redox catalyst for redox-ODH of ethylbenzene. Details related to catalyst preparation and experimental setup are provided in the “Methods” section in the supplementary document (see Supplementary Fig. 1). As shown in Fig. 2a, redox-ODH on fully oxidized (Ca/Mn)_1 − *x*_O@KFeO_2_ can be divided into two regions. Region 1 exhibited high CO_2_ selectivity (29.1%), whereas region 2 exhibited 94.6% styrene selectivity with merely 0.1% selectivity towards CO_2_. A partial reoxidation with air can tune the degree of oxidation for the (Ca/Mn)_1 − *x*_O@KFeO_2_ redox catalyst to take the advantage of the highly selective region 2. As can been seen in Fig. 2b, the nonselective region 1 was near completely eliminated and (Ca/Mn)_1 − *x*_O@KFeO_2_ exhibited excellent ethylbenzene conversion (97%) and styrene selectivity (94.2%) throughout the ODH step. Figure 2c summarizes the redox-ODH performance of (Ca/Mn)_1 − *x*_O@KFeO_2_ as a function of ethylbenzene partial pressure. We note that commercial ethylbenzene DH (DH) process typically operates in a two-stage reactor with interstage reheating and at steam to ethylbenzene molar ratio of ~22:1^8^. The ethylbenzene partial pressure entering the secondary stage of the commercial reactor is around 0.1 atm. At this partial pressure, the single-pass ethylbenzene conversion is thermodynamically limited to 73% at 600 °C, whereas the actual plant performance gives rise to 64% ethylbenzene conversion with 83% selectivity towards styrene (53.1% single-pass yield). In comparison, redox-ODH is not subjected to this equilibrium limitation since in situ combustion of the H_2_ by-product with (Ca/Mn)_1 − *x*_O@KFeO_2_ leads to a thermodynamically favored, exothermic process. As can be seen in Fig. 2c, near 100% conversion of ethylbenzene can be achieved throughout the partial pressure ranges we investigated (up to 0.1 atm). More importantly, styrene selectivity maintained at a high level (94%) with 0.1 atm (industrially comparable) ethylbenzene feed (balance Ar). The experimentally demonstrated redox-ODH yield at 0.1 atm ethylbenzene was 18% higher (absolute basis) than the maximum equilibrium yields for the conventional DH route. Compared to the practical yield in the commercial DH process, redox-ODH demonstrated 38% yield increase (absolute basis) or 72% on a relative basis. In all cases, close to 100% H_2_ were combusted to steam, ensuring autothermal operation while providing a protective atmosphere to maintain oxidation state of the catalytic surface without the needs for externally injected steam (Supplementary Fig. 2). Practically speaking, redox-ODH has the potential to operate at 1 atm ethylbenzene feed without steam dilution. The long-term stability of the redox catalyst was verified, as summarized in Fig. 2d. Aside from a gradual increase in styrene selectivity over the first 50 cycles, stable and satisfactory redox catalyst performances were observed over the 100 cycle continuous testing with complete reoxidation of the redox catalyst. Coke formation was <5% and was completely removed during the reoxidation step. It is noted that the redox-ODH performance relies on the participation of lattice oxygen species and deeply reduced (lattice oxygen-deprived) (Ca/Mn)_1 − *x*_O@KFeO_2_ exhibits much lower ethylbenzene conversion and H_2_ by-product conversion (Supplementary Fig. 3). Further characterization of the redox catalyst and the reaction mechanism is discussed next.

### Redox catalyst characterizations

**Fig. 3:**
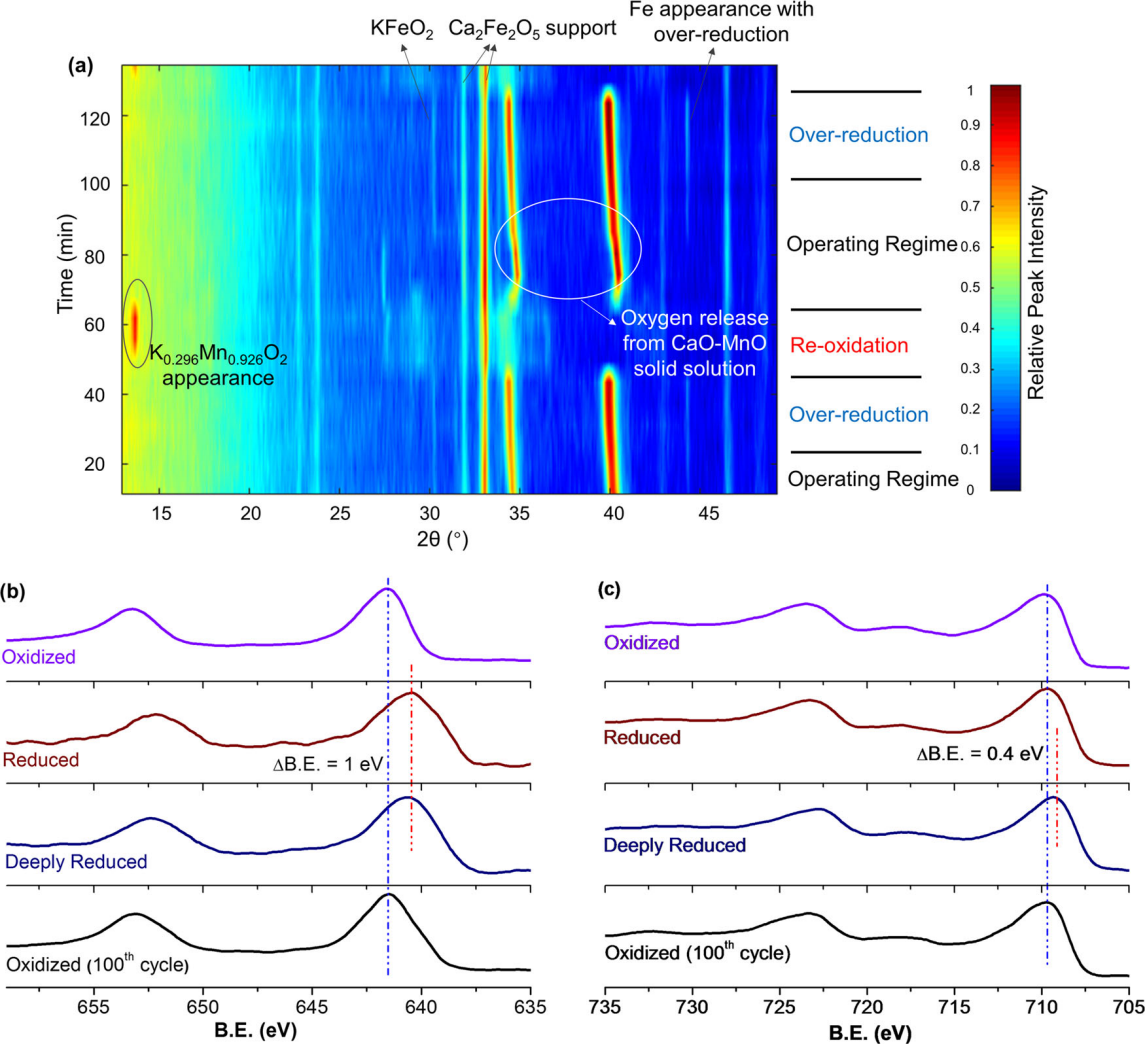
Structural evolution of the redox catalyst. **a** In situ XRD under cyclic ethylbenzene ODH and air reoxidation steps at 600 °C; and XPS of Mn 2*p* (**b**) and Fe 2*p* (**c**) for redox catalysts at different reaction stages.

Figure 2a indicates that the degree of reoxidation of (Ca/Mn)_1 − *x*_O@KFeO_2_ has a significant impact on the styrene selectivity. Since the redox catalyst’s phases, surface elemental compositions, and surface structures undergo dynamic changes in the redox-ODH scheme, detailed understanding of such changes can reveal important mechanistic insights. Figure 3a shows the dynamic phase change observed with in situ X-ray diffraction (XRD) under cyclic ethylbenzene ODH and air reoxidation steps at 600 °C. Under the styrene-selective region (region 2), the primary phases observed include a CaO–MnO solid solution phase and a potassium ferrite (KFeO_2_) phase. The CaO–MnO solid solution, with cation defects, continues to release lattice oxygen during the ODH step, as indicated by the continuous peak shift (from 40.5 to 39.9° and from 34.9 to 34.4°). Over-reduction of (Ca/Mn)_1 − *x*_O@KFeO_2_ formed metallic Fe, which can be reversibly incorporated back into the redox catalyst. On the other hand, complete oxidation of (Ca/Mn)_1 − *x*_O@KFeO_2_ led to the dynamic exchange of cations between the CaO–MnO solid solution and KFeO_2_, forming two new phases, that is, Ca_2_Fe_2_O_5_ and K_0.296_Mn_0.926_O_2_. KFeO_2_ is still observed in the fully oxidized redox catalyst. Detailed XRD peak assignment for (a) fully oxidized redox catalyst and (b) redox catalyst under redox-ODH operating regime are shown in Supplementary Fig. 4a, b. Transmission electron microscopy with energy-dispersive X-ray spectroscopy (TEM-EDS) further confirmed that Ca and Mn were segregated in separate phases for the fully oxidized redox catalyst (Supplementary Fig. 5). Partial reoxidation, however, prevents the formation of K_0.296_Mn_0.926_O_2_ (Supplementary Fig. 4c). The dynamic phase change observed with in situ XRD is consistent with the Mn and Fe oxidation state measurements obtained from X-ray photoelectron spectroscopy (XPS) for the redox catalyst at different reaction stages. Based on stoichiometry and literature^46,47^, Mn oxidation state in K_0.296_Mn_0.926_O_2_ is +4, whereas in CaO–MnO it is between +2 and +3. As shown in Fig. 3b, it is observed in Mn 2*p* XPS that peaks shift to lower binding energy (BE) by 1 eV during the reaction stage. This corresponds to the phase transformation from K_0.296_Mn_0.926_O_2_ (Mn^4+^) to CaO–MnO (Mn^3+/2+^). The XPS peak fittings on Mn 2*p*_3/2_ peaks further agreed with this oxidation change, as shown in Supplementary Fig. 6. In comparison, Fe 2*p* peaks stayed at the same BE for the oxidized and reduced samples (Fig. 3c) and the oxidation state remained at Fe^3+^ (see Supplementary Fig. 6 for detailed peak fitting on Fe 2*p*_3/2_). These results indicate that, within the operating regime, Fe oxidation state stayed constant at +3 resulting from the “sacrificial” reduction of Mn^3/4+^. Given that KFeO_2_ with Fe oxidation state being +3 is known to be the active phase for DH of ethylbenzene^48^, the presence of Mn in the redox catalyst not only helps to maintain Fe at a desirable oxidation state but also provides the lattice oxygen donation/storage capabilities. These results are further confirmed by Mössbauer spectroscopy measurements (Supplementary Fig. 7), where fully oxidized and reduced (Ca/Mn)_1 − *x*_O@KFeO_2_ under operating scheme exhibited consistent Fe oxidation states. It is also noted that deep reduction of (Ca/Mn)_1 − *x*_O@KFeO_2_, which is outside our typical operating regime, reduces Fe oxidation state as seen by the downward shift in BE by 0.4 eV. This is consistent with the observation of metallic iron under XRD and is accompanied with a decrease in catalytic activity due to the lack of active lattice oxygen for redox-ODH (Supplementary Fig. 3). The majority of the iron cations, however, remain at +3 state.

**Fig. 4:**
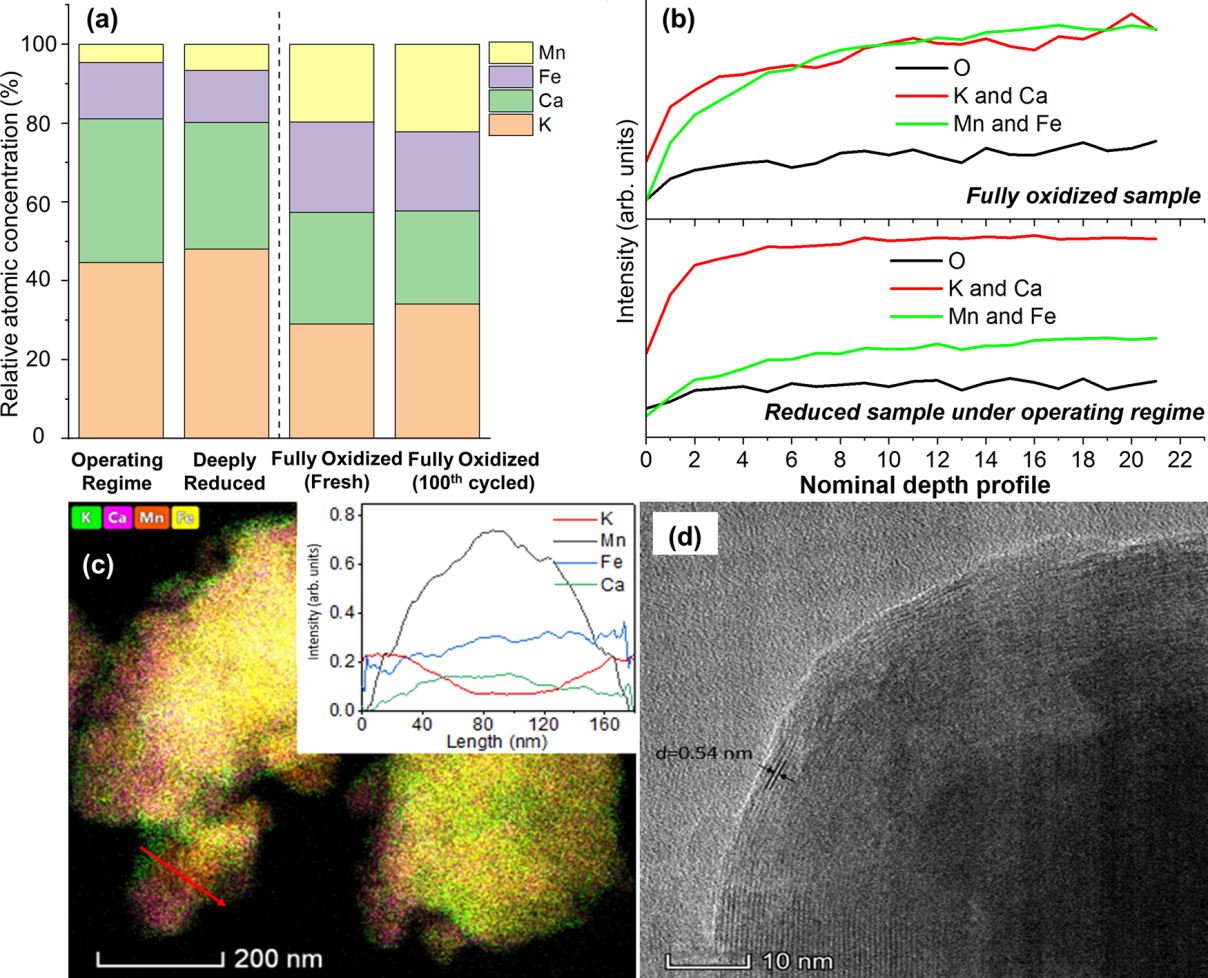
Surface and morphology characterizations of the redox catalyst. **a** XPS surface elemental composition, **b** low-energy ion scattering (LEIS) depth profiling, **c** EDS and line scan, and **d** high-resolution TEM of surface structure.

Besides the dynamic phase/oxidation state changes, (Ca/Mn)_1−*x*_O@KFeO_2_ also undergoes dynamic surface composition and structure changes during redox-ODH of ethylbenzene. The surface elemental composition change, determined by XPS, is illustrated in Fig. 4a. XPS shows that the surface of fully oxidized (Ca/Mn)_1 − *x*_O@KFeO_2_ contains a significant amount of Mn (23%) and Fe (20%). On the other hand, reduced redox catalyst is highly K-enriched and the presence of surface Mn was reduced by ~5-folds. The surface enrichment of K on reduced samples was further confirmed with LEIS spectroscopy, which can detect the composition of topmost surface layers of a redox catalyst^49^. LEIS confirmed that the reduced samples are enriched with K and surface Mn/Fe are significantly suppressed (Fig. 4b). These were also confirmed with TEM-EDS in Fig. 4c, d. Consistent with in situ XRD results, EDS on fully oxidized sample showed a segregation between Ca and Mn, with no surface enrichment feature clearly observed (Supplementary Fig. 5). In comparison, the reduced sample, within the operating range of redox-ODH, showed a surface enrichment of K and a bulk enrichment of Mn as shown by the EDS line scan (Fig. 4c). High-resolution TEM showed that the surface structure of the partially oxidized sample belongs to (101) plane of crystalline KFeO_2_ phase, according to the *d*-spacing (Fig. 4d). This shows that a core-shell structure is formed on redox catalyst under its operating condition, where the core is MnOx-enriched ((Ca/Mn)_1 − *x*_O) and the shell is a layer of KFeO_2_.

### Reaction mechanism study

**Fig. 5:**
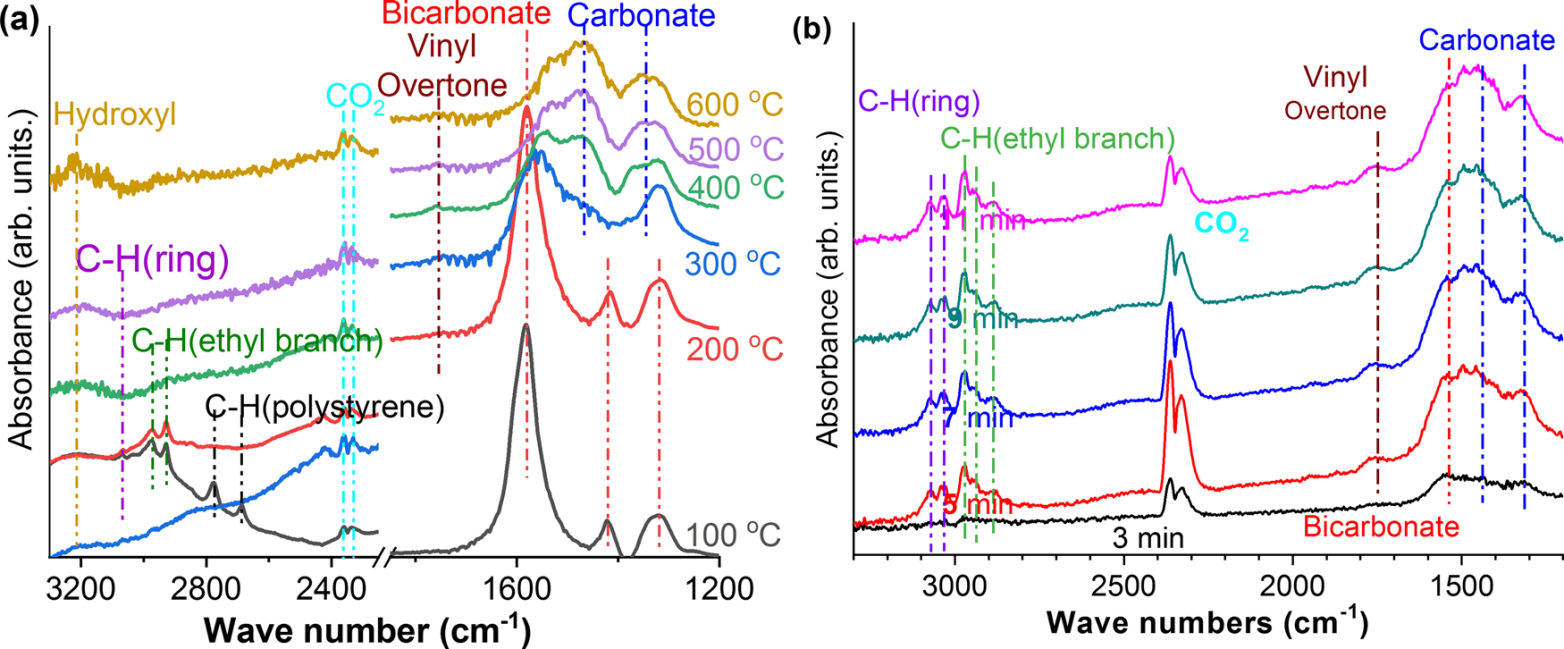
In situ IR spectra analysis. In situ IR spectra collected during **a** TPSR on (Ca/Mn)_1 − *x*_O@KFeO_2_ from 100 to 500 °C and **b** isothermal ethylbenzene ODH on fully oxidized (Ca/Mn)_1 − *x*_O@KFeO_2_ at 500 °C.

Ethylbenzene Fourier transform infrared spectroscopy (FTIR) with both temperature-programmed surface reaction (TPSR) and isothermal mode were further conducted to determine the surface properties of the core-shell structured redox catalyst. As can be seen in Fig. 5a for TPSR experiment on the operating range (Ca/Mn)_1 − *x*_O@KFeO_2_, chemisorption of ethylbenzene on the catalyst surface leads to the formation of bicarbonate and a notable amount of polystyrene species at 100 °C, which subsequently disappeared at 200 °C. As reported by Wu and coworkers^13^ on a high-surface-area CeO_2_ catalyst^13^, the polystyrene species are precursors for coke formation. The absence of the polystyrene peak at operating temperatures for (Ca/Mn)_1 − *x*_O@KFeO_2_ indicates a lower tendency for coke formation on the redox catalyst. The peak around 1600 cm^−1^ was characterized to be bicarbonate instead of deposited carbon based on fitting of other peaks and this bicarbonate was converted to carbonate at elevated temperatures^50^. In addition, a vinyl overtone peak appeared at 1800 cm^−1^, which corresponds to the formation of surface styrene species^13^. Isothermal FTIR was conducted in Fig. 5b, with continuous ethylbenzene feed on fully oxidized (Ca/Mn)_1 − *x*_O@KFeO_2_. Both polystyrene and coke peaks were absent, whereas styrene was formed based on the presence of the vinyl peak. The trend for CO_2_ peaks matched well with the reactivity performance shown in Fig. 2a, where CO_2_ selectivity is high during the oxidized stage (region 1), whereas styrene selectivity dominates within the operating regime (region 2). A stable carbonate species was still observed but it does not appear to affect the catalyst activity. The formation of carbonate species on the ethylbenzene-contacted redox catalyst was also confirmed by XPS peak fittings of the O 1*s* peak, as shown in Supplementary Fig. 8. The FTIR results indicate that surface polystyrene species formed on (Ca/Mn)_1 − *x*_O@KFeO_2_ were further decomposed into styrene rather than coke and the redox catalyst was largely immune to coke formation. This is consistent with the product distribution results in Fig. 2a. Raman was further conducted on used redox catalyst after ethylbenzene ODH and confirmed the minimal amount of carbon deposition (Supplementary Fig. 9).

**Fig. 6:**
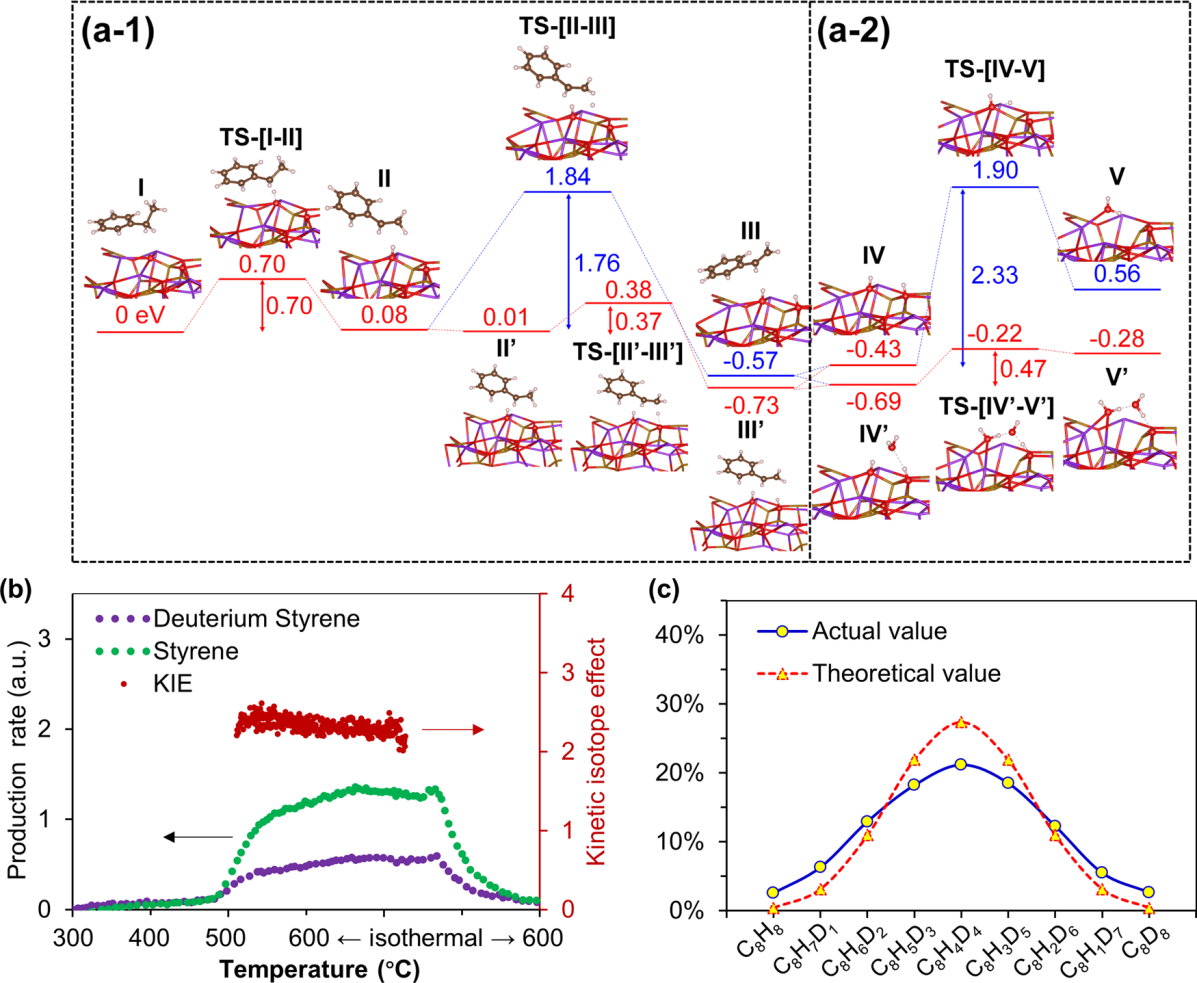
Reaction pathway and rate-limiting step. **a** Computed energy potential profiles of each elementary step for the ethylbenzene ODH and water-assisted proton transfer and water formation (**a-1** corresponds to dehydrogenation steps and **a-2** corresponds to water formation and desorption steps). **b** Kinetic isotope effect study with both temperature-programmed reduction and isothermal reduction of the redox catalyst using ethylbenzene and deuterium ethylbenzene. **c** Styrene isotopes distribution from isothermal ODH using co-feed of ethylbenzene and deuterium ethylbenzene.

Based on the structural information obtained, it can be concluded that a layer of KFeO_2_ predominantly covers the redox catalyst surface, whereas lattice oxygen from the (Ca/Mn)_1 − *x*_O solid solution reversibly donates its lattice oxygen for the redox-ODH reaction. DFT simulations were hence conducted on KFeO_2_ (124) surface, which is one of its most stable crystal surfaces^51^. The Cartesian coordinates of each important transition state were attached in the Vienna ab initio simulation package POSCAR format in the Supplementary material. Various orientations of ethylbenzene adsorption on KFeO_2_ were considered and the most stable adsorbed configuration was determined (Supplementary Fig. 10), shown as configuration I in Fig. 6a. C–H dissociation from α-C is considered as the first hydrogen dissociation step due to its much lower (thermodynamic) reaction energy (0.08 eV) than the H abstraction from β-C (0.91 eV) (Supplementary Fig. 11). β-H dissociation is considered as the second reaction step. Compared with direct H transfer, slightly shifting of the C_8_H_9_ toward the target lattice oxygen (from configuration II to II′ in Fig. 6a) can reduce the distance between the β-H and the target oxygen from 3.55 to 2.41 Å. This largely reduces the steric hindrance and thereby lowering the barrier for β-H abstraction from 1.76 to 0.37 eV with a transition state of ethyl branch attached in parallel to the KFeO_2_ surface (configuration TS-[II-III] in Fig. 6a). Electron density shifts in the transition state structures of α and β hydrogen abstraction are depicted in Supplementary Fig. S12a, b, showing that electrons tend to transfer from the C–H bonds to O–H bonds, which correspond to the trends of C–H bonds cleavage and O–H bonds formation. DFT calculation also indicated that a transition state via C–H dissociation from the benzene ring is not likely (Supplementary Fig. 13). The computed activation energies of α and β hydrogen abstraction (0.70 and 0.37 eV) are much lower than those reported over other catalyst surfaces, such as V_2_O_5_ (001) (1.96 and 2.23 eV)^52^, CeO_2_ (111) (1.70 and 0.84 eV)^53^, and zirconium vanadate (2.48 and 1.23 eV)^54^, showing the advantages of our proposed catalyst. Following the α and β hydrogen abstraction, the formed styrene readily desorbs from the catalyst surface with highly favorable energetics. The low tendency for styrene adsorption on the surface also explains the excellent selectivity observed experimentally. This finding is consistent with literatures, where KFeO_2_ was identified as the active phase for ethylbenzene DH in styrene ^5,48,55,56^.

Meanwhile, DH of ethylbenzene to styrene leaves two hydroxyls (-OH) on the KFeO_2_ surface. To complete the ODH reaction, a proton from one -OH needs to migrate to an adjacent -OH site to form water, which then desorbs from the surface. However, this process is highly endothermic with a prohibitively high barrier of 2.33 eV. Further investigation indicated that the formation of water from unselective combustion of hydrocarbons and/or in situ selective combustion of hydrogen further facilitates ODH activity, as indicated in Fig. 6a–2. As can be seen, the energy barrier for water formation decreased to 0.47 eV under a water-assisted proton transfer mechanism. Here, a hydrogen bond formed between a hydroxyl and water molecule. The hydrogen bond, formed in situ under redox-ODH, facilitates proton transfer on the KFeO_2_ surface, leading to more than 4-folds decrease in the energy barrier for water formation. A similar water-assisted proton transfer phenomena were reported by Merte et al.^57^ as determined by DFT and scanning tunneling microscopy on a FeO thin film. We also explored the effect of water on α and β hydrogen abstraction and did not observe any enhancement effect, indicating that water does not participate in these steps. As such, the overall reaction pathway is summarized in Fig. 6a. The key findings include: (1) α-hydrogen abstraction is the rate-limiting step for ethylbenzene ODH; (ii) water molecules formed from in situ combustion of hydrogen plays an important role in facilitating the ODH reaction, highlighting the importance of the (Ca/Mn)_1 − *x*_O solid solution in terms of providing active lattice oxygen for hydrogen combustion; (iii) styrene molecules readily desorbs from the KFeO_2_ surface, supporting the high catalytic activity/selectivity of (Ca/Mn)_1 − *x*_O@KFeO_2_ and the importance of maintaining a KFeO_2_ terminated surface.

The DFT calculation results are further supported by experimental data. For instance, isotope exchange between H-hydroxylated (Ca/Mn)_1 − *x*_O@KFeO_2_ and deuterated ethylbenzene in FTIR-TPSR experiments indicated that only the Ds from the ethyl branch of ethylbenzene were activated/exchanged <200 °C (Supplementary Fig. 14). This confirms that α and β hydrogen abstraction being the critical first steps for ethylbenzene ODH. To further determine the rate-limiting step, kinetic isotope effect (KIE) for the ODH reaction rates of ethylbenzene (C_8_H_10_) and deuterated ethylbenzene (C_8_D_10_) were measured (Fig. 6b). The KIE values were determined to be between 2.12 and 2.62. This confirms that the C–H bond activation on the ethyl branch of ethylbenzene being the rate-limiting step^58^ and is consistent with the DFT results. Another interesting experimental finding via co-feeding of C_8_H_10_ and C_8_D_10_ (Fig. 6c) is that the resulting styrene isotopes with different amount of D-substitution almost follows a Gaussian distribution. This is only possible in the case of a perfect isotope scrambling, where all H in C_8_H_10_ have the same chance of being exchanged into D. This confirms that: (a) as-formed styrene molecules are absorbed parallel to the KFeO_2_ surface in a conjugated electronic structure (consistent with DFT calculated configuration III in Fig. 6a-1); (b) water molecules are likely to facilitate proton exchange. The effect of water on proton exchange (and hence ethylbenzene conversion and water formation/desorption) was further confirmed with KFeO_2_ without (Ca/Mn)_1 − *x*_O. Without co-injection of water, stand-alone KFeO_2_ cannot donate its lattice oxygen for in situ water formation, showed low ethylbenzene conversion (44.3%) and the H_2_ by-product was not effectively oxidized to water (Supplementary Fig. 15). Overall, these mechanistic findings, supported by both experiment and DFT calculations, provide important insights for redox catalyst design and optimizations.

### Process performance and impacts

To quantify the practical impacts of the (Ca/Mn)_1 − *x*_O@KFeO_2_ redox catalyst in the context of the redox-ODH scheme, detailed ASPEN Plus simulations were carried out to simulate both the commercial DH and redox-ODH schemes. The process configuration and operating conditions for commercial ethylbenzene DH process were documented in detail by Luyben^8^. As detailed in the Supplementary document, our simulation accurately reproduced the performance of the commercial DH process (Case 1 in the Supplementary document). Two cases for the redox-ODH process were also analyzed: (1) The base case (Case 2) evaluates the redox-ODH process using the experimental performance of the (Ca/Mn)_1 − *x*_O@KFeO_2_ redox catalyst at 0.1 atm ethylbenzene feed pressure, assuming 90% steam dilution; (2) An optimal case (Case 3), also using the experimental performance but assuming no steam dilution, was considered as well. We note that ethylbenzene was injected in dry conditions without steam dilution in all our experimental cases. These results support the potential feasibility of Case 3, that is, complete avoidance of steam usage in redox-ODH of ethylbenzene.

**Fig. 7:**
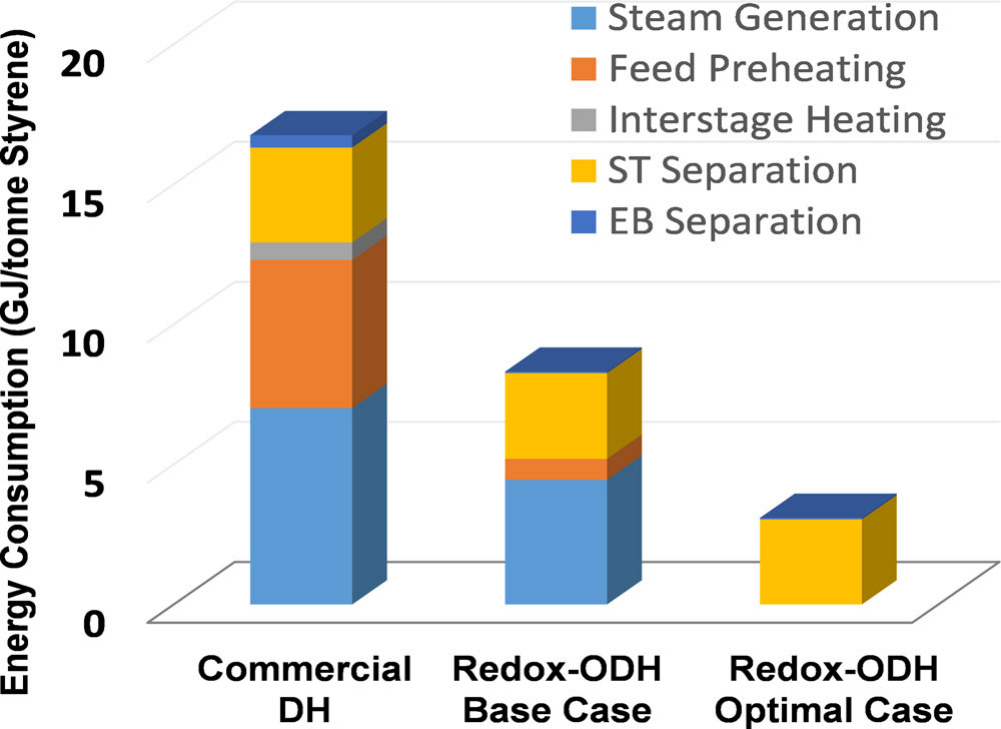
Process analyses. Energy intensities of commercial DH and redox-ODH for styrene production based on ASPEN Plus simulation.

Figure 7 summarizes the process performance of the three cases. As can be seen, 50–82% energy savings can be anticipated from the redox-ODH cases, with steam generation (including heating/reheating) being the main driver for energy consumptions. Given that the energy is provided by fossil fuels in styrene production, CO_2_ emission can be reduced by up to 79% with the redox-ODH approach (accounting for offset with hydrogen by-product in commercial DH). The significant energy/emission savings by redox-ODH are primarily driven by the improved energy management and reduced steam usage. In the commercial DH process, a large steam dilution ratio (~22:1) is used. Moreover, steam needs to be superheated to provide the heat for the highly endothermic DH reaction. As a result, 12.3 GJ of energy is required for steam generation and reheating in order to produce each metric ton of the styrene product. In contrast, the base case redox-ODH significantly reduces steam usage (9:1 dilution ratio). Moreover, the exothermic ODH reaction offsets the energy consumption for steam heating/reheating, leading to a net energy consumption of 5.2 GJ/ton of styrene. This energy consumption can be eliminated given that redox-ODH does not need steam co-feed in principle. Besides the energy savings for steam generation, redox-ODH is more efficient in all the other key process steps. Particularly, the high product selectivity and yield leads to reduced amount of recycling as well as decreased energy consumptions for ethylbenzene and styrene separations with a smaller number of separation trays. The high styrene selectivity in redox-ODH provides an overall styrene yield of 91.4%, or 38% higher than that in the commercial DH process (53.1%). These process analysis results clearly demonstrate the advantages of the (Ca/Mn)_1 − *x*_O@KFeO_2_ redox catalyst and the redox-ODH process scheme.

The current study proposed and demonstrated a redox-ODH approach that autothermally converts ethylbenzene to styrene with 91.4% overall yield and 50–82% energy and CO_2_ emission savings, when compared to the commercial styrene DH process. To facilitate ethylbenzene conversion, a tailored, multifunctional redox catalyst, which acts as a heterogeneous catalyst, an oxygen separation agent, and a selective oxidation material, was developed and characterized. In redox-ODH, the (Ca/Mn)_1 − *x*_O@KFeO_2_ redox catalyst not only catalyzes ethylbenzene conversion but also donates its active lattice oxygen for selectively hydrogen combustion in a recyclable manner. Ninety-seven percent ethylbenzene single-pass conversion, 94.2% styrene selectivity, and ~100% H_2_ conversion was achieved without co-feeding steam. Detailed characterizations indicate that the redox catalyst undergoes dynamic bulk and surface structural changes. Under the working conditions, (Ca/Mn)_1 − *x*_O@KFeO_2_ is terminated with a KFeO_2_ rich surface, which is responsible for catalyzing ethylbenzene conversion. Meanwhile, its bulk phase is composed of a cation defected CaO–MnO solid solution that is responsible for reversible lattice oxygen donation (in the ODH step) and uptake (in the reoxidation step). The lattice oxygen donation was facilitated by Mn +3 to +2 transition and the “sacrificial” reduction of Mn cations helps to stabilize the oxidation state of Fe, which remains at its most catalytically active +3 state. Moreover, the facile donation of lattice oxygen also retards coke formation. Through DFT calculations along with experimental validations, a detailed reaction pathway for the redox-ODH reaction on the KFeO_2_ surface was established. It was determined that α hydrogen abstraction is the rate-limiting step for ethylbenzene ODH and water molecules formed in situ significantly lowers the energy barrier for hydrogen combustion via a water-assisted proton transfer mechanism. Moreover, styrene molecules readily desorb from the KFeO_2_ surface, ensuring high product selectivity. These findings, which unveil the roles of the KFeO_2_ surface, (Ca/Mn)_1 − *x*_O bulk phase, and their synergistic effects, provide important mechanistic insights for designing effective redox catalysts for alkylbenzene conversions. Finally, detailed process simulations based on experimental data indicate up to 82% reduction in energy consumption and 79% decrease in CO_2_ emissions. This potentially translates into ~14 million tonnes of CO_2_ emission reductions each year for this important yet carbon-intensive industrial process.

## Methods

### Redox catalyst synthesis

The (Ca/Mn)_1 − *x*_O@KFeO_2_ redox catalyst was prepared in two steps. The (Ca/Mn)_1 − *x*_O substrate was synthesized by a modified Pechini method. Mn(NO_3_)_2_·4H_2_O (8.26 g, 97.0%, Sigma-Aldrich) and Ca(NO_3_)_2_·4H_2_O (8.77 g, 99.0%, Sigma-Aldrich) were dissolved in deionized water with stirring at 30 °C. Citric acid (26.87 g, 99.5%, Sigma-Aldrich) was then added into the solution. Next, 11.7 ml ethylene glycol (99.8%, Sigma-Aldrich) was added to the solution. The obtained solution was kept at 80 °C with stirring until a gel formation. The gel was then transferred to an oven for drying at 130 °C for 24 h. The precursor was finally calcined in a furnace at 950 °C for 12 h (5 °C/min ramping rate) under continuous airflow. The sample obtained after calcination was crushed into 20–40 mesh. (Ca/Mn)_1 − *x*_O@KFeO_2_ was synthesized via wet impregnation by using (Ca/Mn)_1 − *x*_O as the substrate. Weigh 4 g of the (Ca/Mn)_1 − *x*_O substrate synthesized and put into a glass beaker. Weigh 0.91 g of KNO_3_ (99.9%, Sigma-Aldrich) and 18.10 g of Fe(NO_3_)_3_·9H_2_O (98%, Sigma-Aldrich) into another glass beaker and add deionized water to dissolve these. The formed solution was then added into the (Ca/Mn)_1 − *x*_O-containing beaker and stirred at 80 °C until dried. The obtained mixture was calcined in a furnace under air continuous flow at 650 °C for 3 h, and then crushed into 20–40 mesh as final (Ca/Mn)_1 − *x*_O@KFeO_2_ redox catalyst. Stand-alone KFeO_2_ was synthesized by adding equal molar amounts of KNO_3_ and Fe(NO_3_)_3_·9H_2_O into a glass beaker. Specifically, 3.18 g KNO_3_ and 12.73 g Fe(NO_3_)_3_·9H_2_O were added into a glass beaker, and water was then added into the beaker. The solution was stirred at 80 °C until dried. The resulting solids were calcined in a furnace under air continuous flow at 650 °C for 3 h to obtain stand-alone KFeO_2_.

### Ethylbenzene redox-ODH reactivity test

Ethylbenzene redox-ODH was conducted via a cyclic experiment. Ethylbenzene ODH step was conducted first. In this step, 0.5 g of redox catalyst was loaded into a fixed bed quartz U-tube reactor with ID of 1/8 in. The U-tube reactor was heated via a furnace equipped with a K-type thermocouple. Typical ethylbenzene ODH reaction was conducted at 600 °C. Ethylbenzene vapor was introduced into the U-tube reactor via a bubbler setup in Supplementary Fig. 1. The bubbler was heated in an oil bath. Ar gas (25 ml/min) was introduced into the bubbler, and the exit gas was Ar/ethylbenzene mixture. The ethylbenzene partial pressure was adjusted by changing the temperature of the oil bath. For example, the oil bath was set at 25 °C to obtain an ethylbenzene partial pressure of ~0.01 atm. The ethylbenzene ODH step lasted for 30 min. The products were detected and quantified via a downstream quadruple mass spectroscopy (QMS, MKS Cirrus II) or gas chromatography (Agilent 7890A). After the ethylbenzene ODH step, the U-tube reactor was purged for 20 min. Then, simulated air (25 ml/min, 20% O_2_ balanced in Ar) was introduced into the heated U-tube reactor at 600 °C for the air reoxidation step. The air reoxidation step lasted for 5 min for a redox catalyst full reoxidation, and for 2 min for a redox catalyst partial reoxidation.

### Catalyst characterization

The exothermicity and the weight change of H_2_/O_2_ redox cycles with (Ca/Mn)_1 − *x*_O@KFeO_2_ redox catalyst were measured on a thermogravimetric analyzer (TGA) equipped with differential scanning calorimetry. Approximately 50 micrograms of redox catalysts were loaded into the crucible cell of the TGA (TA instrument). Then, the TGA cell was heated to 600 °C with 10 °C/min under Ar. H_2_/O_2_ redox cycles were conducted under 600 °C. First, 10% H_2_ (balanced in Ar) was introduced for 5 min as the redox catalyst reduction step. Then, the cell was purged with pure Ar for 5 min. After that, 20% O_2_ (balanced in Ar) was introduced for 5 min as the redox catalyst reoxidation step. Another 5 min Ar-purging step was followed after that to finish one redox cycle. The redox cycle was repeated for ten times and a stabilized exothermicity/weight change was achieved.

In situ XRD experiments were conducted on an Empyrean X-ray diffractometer equipped with an Anton-Parr XRK-900 reactor chamber. A scanning range of 10 − 50° (2*θ*) was used to obtain XRD patterns by using Cu Kα (*λ* = 0.1542 nm) radiation operating at 45 kV and 40 mA. To determine the phase behavior and stability of the catalyst, a ethylbenzene temperature-programmed reduction (TPR) in the range 300–650 °C (ethylbenzene-TPR) and isothermal redox at 600 °C were performed. Before the redox, fresh catalyst was loaded into the chamber and scanned to obtain a pattern at room temperature. Before the experiment, the reaction system (reactor chamber, ethylbenzene bubbler, and connected gas line) are sufficiently purged to remove air and possible contaminants. In ethylbenzene-TPR experiment, the reactor chamber was first heated to 300 °C under a flow of nitrogen at a flow rate of 25 ml/min. Prior to the ethylbenzene-TPR, the nitrogen was switched to ethylbenzene bubbler at room temperature (25 °C) to generate a ethylbenzene/nitrogen flow with a ethylbenzene partial pressure of ~1%. The sample was heated in ethylbenzene/nitrogen in the range 300–650 °C at a rate of 2 °C/min. Afterwards, the temperature decreased to 600 °C at a rate of 10 °C/min and then kept at 600 °C for 48 min with the continuous ethylbenzene/nitrogen flow. After the reduction, the atmosphere was purged by pure nitrogen for 30 min and reoxidized by air to remove the possible adsorbed hydrocarbons and coke. In the isothermal redox experiment, the sample was heated to 600 °C under the flow of nitrogen at a flow of 25 ml/min. An ethylbenzene/nitrogen flow with an ethylbenzene partial pressure of ~1% was introduced to the XRD cell as the ethylbenzene-TPR step. After the ethylbenzene-TPR step lasted for 30 min, the cell was then purged by pure nitrogen for 18 min. Thereafter, the atmosphere was changed to air tor reoxidize the reduced sample at the same flow rate. Two redox cycles were proceeded in the reactor. Nitrogen was used to purge the reaction system between two cycles. XRD scans were taken every 6 min.

XPS was used to analyze surface compositions and valence states of fresh, 3-min prereduced, 20-min reduced, 40-min deep-reduced, reoxidized, and long-term cycled catalysts. The reduced samples with different reduction time and cycled samples were obtained from the isothermal redox at 600 °C at the condition as mentioned in the section of the catalytic evaluation. The reduced samples were transferred to a glove box filled with nitrogen atmosphere to avoid air exposure and sealed in sampling bottles before transporting to the XPS chamber. The sample powder was pressed onto a carbon tape and outgassed at 10^−5^ Torr for overnight before it was introduced into the ultrahigh-vacuum chamber for scanning. The XPS patterns were collected on a PHOIBIS 150 hemispherical energy analyzer (SPECS GmbH) equipped with a non-monochromatic Mg Kα excitation source (1254 eV). The data were analyzed by the CasaXPS program (Casa Software Ltd, UK). The binding energy was calibrated to a C 1*s* line at 284.6 eV. The surface compositions of K, Ca, Fe, and Mn were calculated according to characteristic peak areas and their respective atomic sensitivity factors.

LEIS was conducted at the Surface Analysis Center at Lehigh University with an ION-TOF Qtac^100^ for surface compositional analysis and depth profiling. The samples tested for LEIS were fully oxidized (Ca/Mn)_1 − *x*_O@KFeO_2_ and 20-min reduced (Ca/Mn)_1 − *x*_O@KFeO_2_ under ethylbenzene ODH operating scheme. The sample collection and storage scheme is the same as the abovementioned XPS section. The detection source was 3 keV He^+^ (5 × 10^14^/cm^2^/cycle, 1.5 × 1.5 mm^2^ raster). The sputtering source was 1.0 keV Ar^+^ (1.0 × 10^15^/cm^2^/cycle, 2 × 2 mm^2^ raster. Due to the similar atomic mass, K and Ca and Mn and Fe cannot be separated in LEIS analysis. Thus, K and Ca and Mn and Fe were lumped together in the surface compositional analysis.

Ethylbenzene TPSR with FTIR (TPSR-FTIR) spectroscopy was conducted using a Thermo Fisher Nicolet iS50 FTIR equipped with a DiffusIR sample chamber (Pike Technologies). The sample was first loaded into the in situ cell and purged with Ar at 100 °C to desorb surface CO_2_. Ethylbenzene vapor was then introduced into the in situ cell at 100 °C for 30 min for ethylbenzene surface adsorption, by flowing Ar through an ethylbenzene bubbler located at room temperature. Then, the ethylbenzene vapor flow was stopped, and the in situ cell was purged with Ar at 100 °C for 30 min. Afterwards, the in situ cell was raised from 100 to 600 °C and FTIR scans were taken at 100–600 °C. Isothermal FTIR was conducted at 500 °C. The in situ cell was held at 500 °C under Ar. Then, ethylbenzene vapor was introduced into the in situ cell by flowing Ar through an ethylbenzene bubbler located at room temperature. FTIR scans were taken every 2 min.

Raman was conducted on the (Ca/Mn)_1 − *x*_O@KFeO_2_ catalyst after 20 min ethylbenzene ODH. The instrument was a multiwavelength Raman system. The signal was collected via a customized ellipsoidal mirror and directed by a fiber optics bundle to the spectrograph stage of a triple Raman spectrometer (Acton Trivista 555 from Princeton Instruments). Edge filters (Semrock) were used in front of the ultraviolet–visible fiber optic bundle (Princeton Instruments) to block the laser irradiation. A 442-nm visible laser was used as the excitation beam, which is generated from a HeCd laser (Melles Griot). A ultraviolet-enhanced liquid N_2_-cooled CCD detector (Princeton Instrument) was employed for signal detection. Transmission electron microscopy with energy-dispersive x-ray spectroscopy (TEM-EDX) was conducted with an aberration-corrected scanning transmission electron microscope—Thermo Fisher Titan 80-300. Mössbauer spectroscopy was conducted in Dalian Institute of Chemical Physics. ^57^Fe Mössbauer spectra were collected at room temperature with a constant accelerations mode using ^57^Co γ-quantum source in the Rh matrix. All spectra were computer-fitted with a least-squares fitting procedure to a Lorentzian shape. The isomer shifts were set with respect to α-Fe at room temperature.

### KIE and isotopic exchange experiments

KIEs were calculated as the ratio of normal ethylbenzene consumption rate to deuterium ethylbenzene consumption rate under identical redox-ODH conditions. The ODH experiments over redox catalysts were conducted separately using ethylbenzene and deuterium ethylbenzene. Typically, KIE was determined using lower conversion (<20%). To achieve ethylbenzene conversions <20%, 30 mg of redox catalyst was loaded into a fixed bed quartz U-tube reactor and then the ethylbenzene/Ar (or deuterium ethylbenzene/Ar) mixture (25 ml/min) with an ethylbenzene partial pressure of ~0.01 atm was introduced into the reactor, as described in Supplementary Fig. 1. The ethylbenzene ODH experiments was conducted in ethylbenzene/Ar in the range 300–600 °C at a rate of 10 °C/min. Afterwards, the temperature was kept at 600 °C for 20 min with the continuous ethylbenzene/Ar flow. Similarly, the deuterium ethylbenzene ODH experiments were conducted by using deuterium ethylbenzene/Ar mixture. The products were detected and quantified via either a downstream or an upstream QMS (MKS Cirrus II).

Isotopic exchange experiment over the redox catalyst was conducted in an isothermal ODH using co-feed of ethylbenzene and deuterium ethylbenzene to explore the styrene isotopes distribution. The same experimental setup in Supplementary Fig. 1 was used. To achieve an ethylbenzene conversion close to 100%, 0.5 g of redox catalyst was loaded into a fixed bed reactor. The bubbler was filled by the same volume of ethylbenzene and deuterium ethylbenzene to obtain an equal partial pressure. The ODH reaction was conducted at 600 °C with the continuous normal-deuterium ethylbenzene/Ar flow (25 ml/min) for 20 min. The styrene products were detected and quantified via a downstream quadruple mass spectroscopy (QMS, MKS Cirrus II). Styrene and deuterated styrene were the main products with *m*/*z* in the range 104–112, corresponding to C_8_H_8 − *x*_D_*x*_ (*x* = 0, 1, 2, 3, 4, 5, 6, 7, 8), respectively. The styrene distributions were calculated on the basis of the relative proportion of the signals of C_8_H_8-x_D_x_ from mass spectroscopy since both the styrene and deuterium styrenes have very similar mass spectroscopy response factors.

### DFT simulation

First-principles simulations were performed at the DFT level implemented by the Vienna ab initio simulation package. All-electron projector augmented wave model and Perdew–Burke–Ernzerhof functions were employed. A kinetic energy cutoff of 450 eV was used for the plane-wave expansion of the electronic wave function. The force and energy convergence criterion was set to be 0.01 eV/Å and 10^−5^ eV, respectively. A Gaussian smearing of 0.1 eV was applied for optimization. A *k*-point grid with a 4 × 4 × 2 Gamma-centered mesh for pure KFeO_2_ unit cell and only Gamma mesh for all the surface slab models were chosen for sampling the first Brillouin zone. Due to the complexity of the KFeO_2_ surface structure, it is very difficult to accurately try each magnetic ordering. To make the calculations tractable, here we apply the ferromagnetic state for all the structures given that the tiny energy differences due to different magnetic ordering are negligible compared with ionic migration. The strong on-site Coulomb interaction on the *d*-orbital electrons on the Fe sites was treated with the GGA + U approach. We adopted *U*_eff_ = 4 eV for Hund’s exchange interaction, which has been proved to give reasonable predictions of both geometric and electronic structures in previous works. The computed lattice constants of KFeO_2_ (*a* = 5.68 Å, *b* = 11.38 Å, *c* = 16.07 Å) match well with the experimental values (*a* = 5.61 Å, *b* = 11.21 Å, *c* = 15.26 Å), showing the reliability of the computational settings^1^. The climbing image nudged elastic band method was applied for transition sate optimization. The adsorption energy of ethylbenzene on KFeO_2_ surface is computed with $E_{\mathrm{ads}} = E_{\mathrm{sur}} + E_{\mathrm{EB}} - E_{{\mathrm{sur}} - {\mathrm{EB}}}$, where $E_{\mathrm{sur}}$, $E_{\mathrm{EB}}$, and $E_{\mathrm{sur - EB}}$ are energies of KFeO_2_ surface model, ethylbenzene molecule, and the adsorbed configuration.

## Supplementary information

Supplementary Information

## Data Availability

The data that support the findings of this study are available from the corresponding author on reasonable request.
