# Supplementary material for: A tailored multi-functional catalyst for ultra-efficient styrene production under a cyclic redox scheme
Source: Nat Commun. 2021 Feb 26;12:1329. doi: 10.1038/s41467-021-21374-2 (PMC7910546; doi:10.1038/s41467-021-21374-2)
Supplement: Supplementary file 1 — Supplementary Information [file 41467_2021_21374_MOESM1_ESM.pdf]

A tailored multi-functional catalyst for ultra-efficient styrene production under a cyclic redox scheme

Supplementary information

Xing Zhu[# a,b], Yunfei Gao[# a], Xijun Wang[a], Vasudev Haribal[a], Junchen Liu[a], Luke M. Neal[a], Zhenghong Bao[c], Zili Wu[c], Hua Wang[b], and Fanxing Li[*a]

[a] Department of Chemical and Biomolecular Engineering, North Carolina State University, United States.

[b] State Key Laboratory of Complex Nonferrous Metal Resources Clean Utilization, Faculty of Metallurgical and Energy Engineering, Kunming University of Science and Technology, Kunming 650093, China

[c] Chemical Science Division and Center for Nanophase Materials Sciences, Oak Ridge National Laboratory, Oak Ridge, Tennessee 37831, United States

[#] These people contributed equally to this work.

*email: fli5@ncsu.edu

Supplementary Data, Figures and Tables:

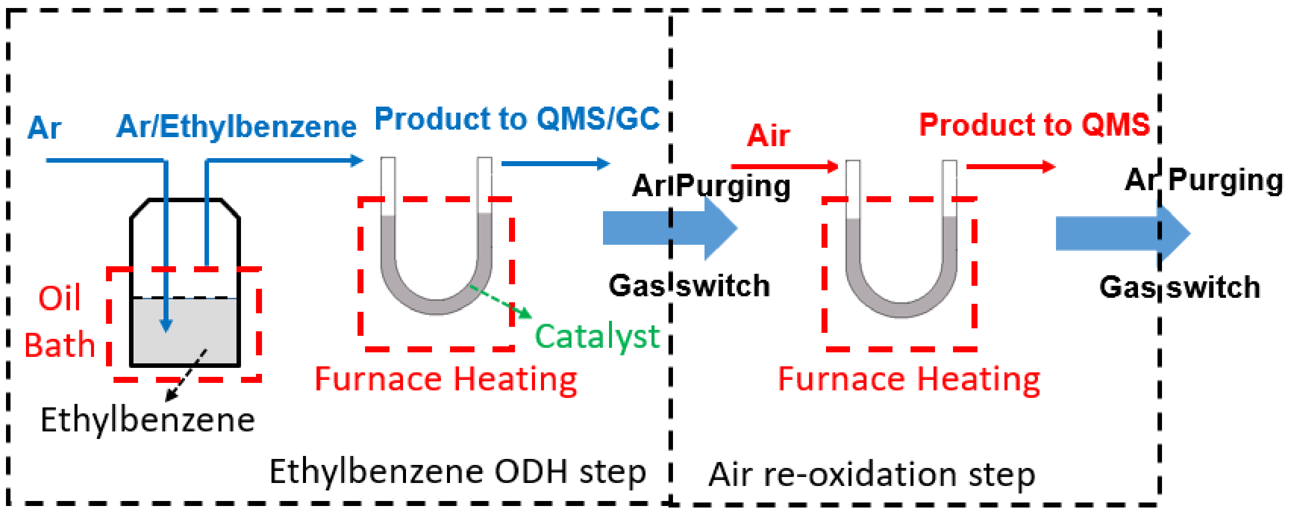

Supplementary Fig. 1 Experimental setup. Scheme for experimental setup of ethylbenzene Redox-ODH

Supplementary Fig. 2 compares the exothermicity of an ethylbenzene direct dehydrogenation (DH) process to the redox-ODH process. The heat of redox reactions and corresponding catalyst weight change of the $(Ca/Mn)_{1-x}O@KFeO_2$ redox catalyst were measured with H_2/O_2 redox cycles under a TGA-DSC, as described in the experimental section. As shown in Supplementary Fig. 2a, TGA-DSC showed that both catalyst reduction step (-25 kJ/mol O consumed, reduced with H_2) and catalyst re-oxidation step (-220.8 kJ/mol O regenerated, oxidized with air) are exothermic. Thus, the overall exothermicity per H_2/O_2 redox cycle is -245.8 kJ/mol O exchanged, which agrees well with the lower heating value of H_2. Meanwhile, thermodynamic calculation indicates that conventional ethylbenzene DH is a highly endothermic process (123.9 kJ/mol ethylbenzene converted to styrene). By adding up this endothermicity for dehydrogenation with the exothermicity of the H_2/O_2 redox cycle, one can obtain the overall (net) exothermicity of the redox ODH process (-121.9 kJ/mol ethylbenzene converted to styrene). This number is consistent with the exothermicity of the conventional O_2-cofeed ethylbenzene ODH process predicted with thermodynamic calculation. Given that: (1) the exothermicity of ethylbenzene conversion step in the redox ODH process can be calculated by adding the endothermicity of conventional DH and the exothermicity of H_2 oxidation to water in H_2/O_2 redox cycles and (2) the exothermicity of catalyst air re-oxidation step in the redox ODH process can be determined by the exothermicity of the catalyst re-oxidation step in H_2/O_2 redox cycles, the calculated exothermicity of each reaction step in the redox ODH process were shown and compared with conventional DH in Supplementary Fig. 2b. As can be seen, Redox-ODH is net exothermic (-121.9 kJ/mol) and can be operated auto-thermally.

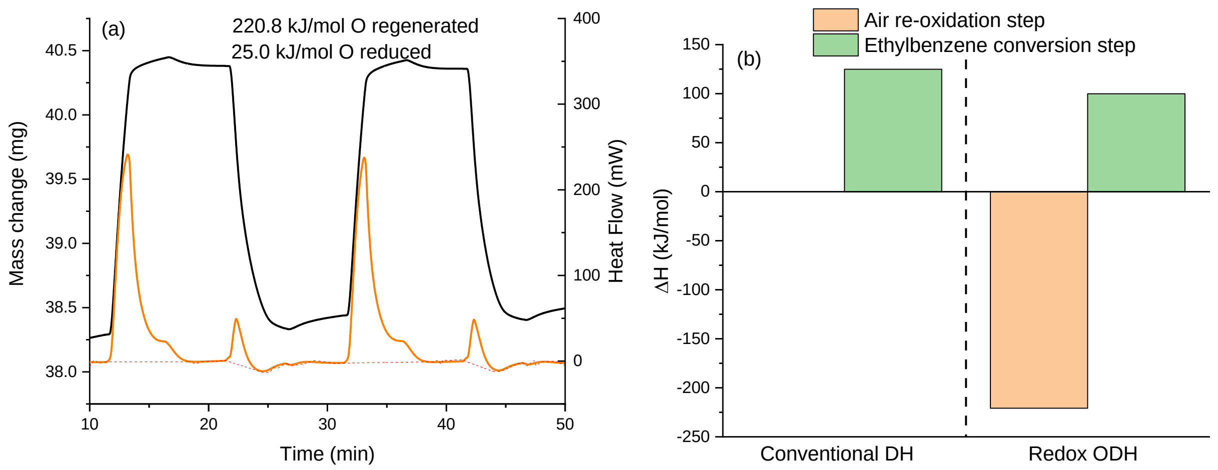

Supplementary Fig. 2 TG-DSC analysis. a TG-DSC profile of $(Ca/Mn)_{1-x}O@KFeO_2$ redox catalyst in the H_2/air redox experiment at 600 °C. Conventional DH case was calculated on the basis of HSC chemistry while the actual cases was analyzed on the basis of H_2-DSC experiments over $(Ca/Mn)_{1-x}O@KFeO_2$ redox catalyst. **b** Comparison of heat of reactions for a conventional dehydrogenation process with the Redox-ODH processes.

Role of lattice oxygen in the redox catalyst was probed by comparing oxidative dehydrogenation behaviors over the $(Ca/Mn)_{1-x}O@KFeO_2$ redox catalysts with 0 wt.% and 90 wt.% available oxygen storage capacity (OSC), as shown in Supplementary Fig. 3. The catalyst with 0 wt.% av. OSC was obtained by using hydrogen to reduce the sample until no water formed at 600 °C. As shown in Supplementary Fig. 3a, the hydrogen conversion is very low (~5%) due to the lack of active lattice oxygen. As a result, the ethylbenzene conversion is limited to 50%. As a comparison, a nearly 100% conversion of hydrogen to water was achieved in redox catalyst with 90 wt.% av. OSC and the corresponding ethylbenzene conversion is as high as 93.2% (Supplementary Fig. 3b). The overall styrene yield is twice comparing $(Ca/Mn)_{1-x}O@KFeO_2$ redox catalyst with no active lattice oxygen remaining (0 wt.% OSC). The results reveals that active lattice oxygen species is essential for Redox-ODH.

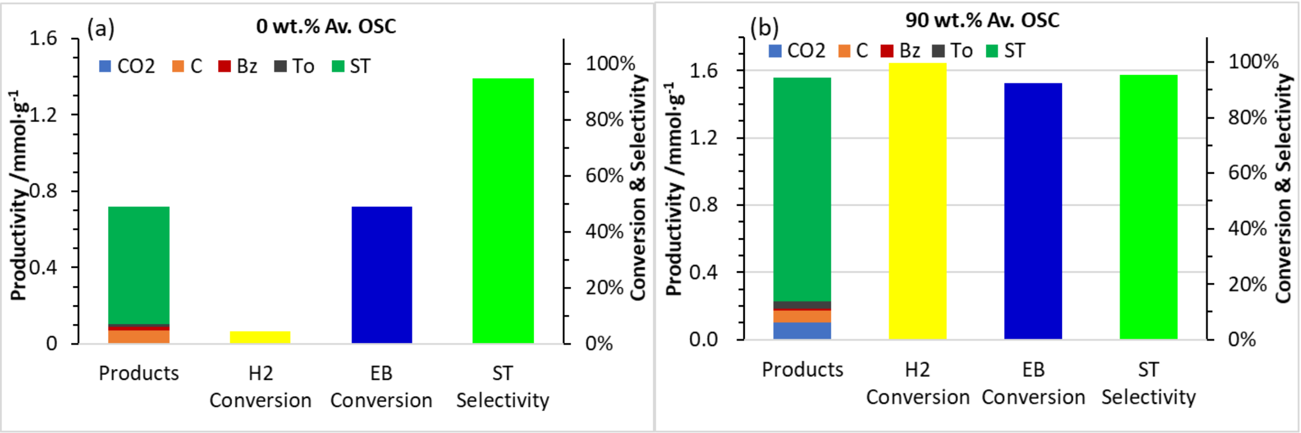

Supplementary Fig. 3 Role of lattice oxygen. Role of lattice oxygen for the oxidative dehydrogenation of ethylbenzene to styrene by comparing the redox catalysts with (**a**) 0 and (**b**) 90% available OSC. EB: ethylbenzene; ST: styrene; To: toluene; Bz: benzene.

Detailed in-situ XRD scan were selected for the fully oxidized, reduced and partially re-oxidized

$(Ca/Mn)_{1-x}O@KFeO_2$ redox catalyst as shown in Supplementary Fig. 4a to S4c respectively. It was observed that there are some low quality peaks between 17 and 30°. These are mostly sub-peaks of the $Ca_2Fe_2O_5$ phase and the low XRD signal is due to the nature of our measurement, which were set at a relatively fast scanning rate (6 mins per scan) to capture the transient phase change of the redox catalyst

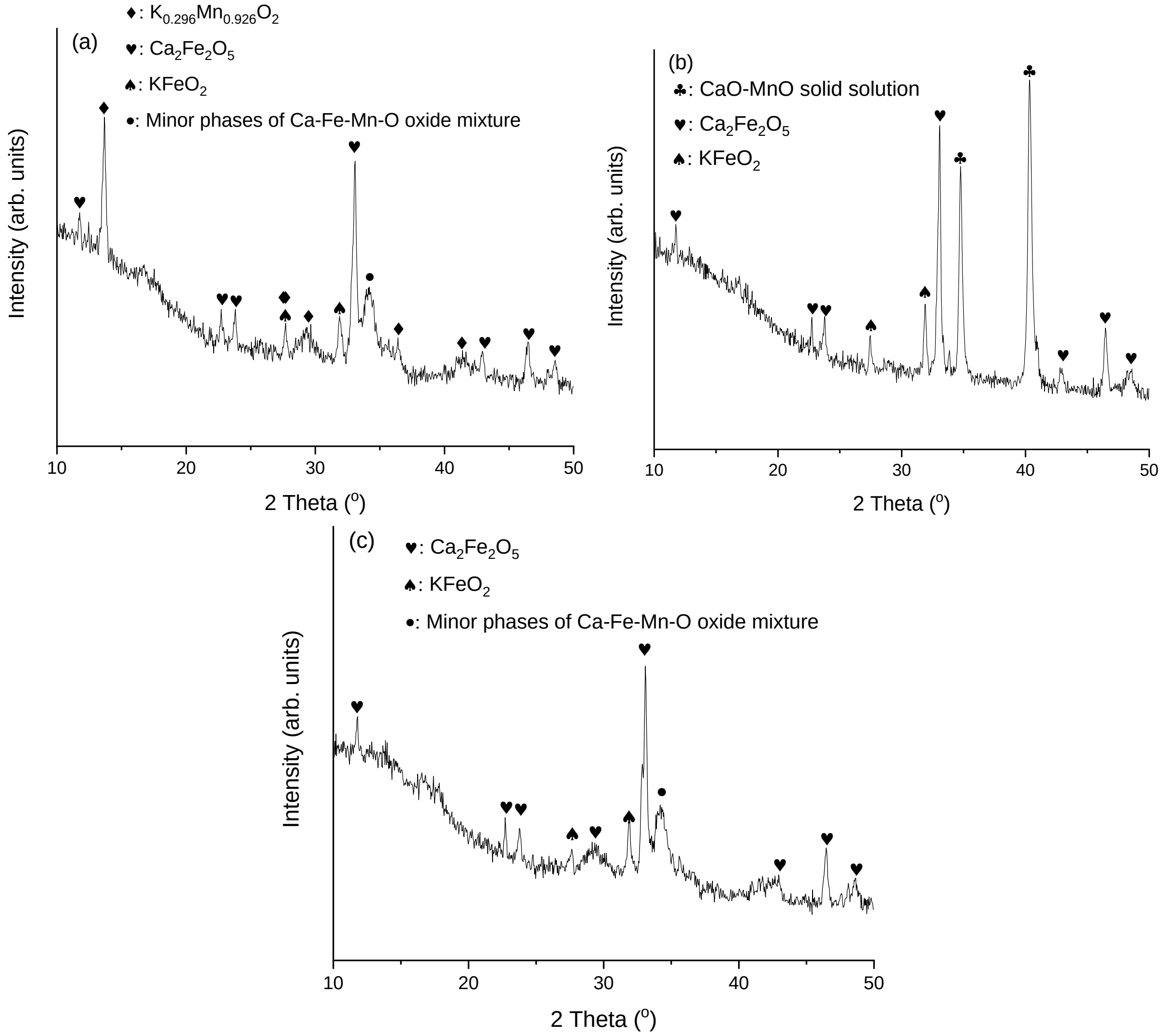

Supplementary Fig. 4 *In-situ* XRD analysis. Detailed phase assignment of *in-situ* XRD of (**a**) fully oxidized redox catalyst, (**b**) reduced redox catalyst under operating regime and (**c**) redox catalyst with partial re-oxidation

TEM-EDX of fully oxidized redox catalyst. The results in Supplementary Fig. 5 indicate that Ca and Mn largely remain in separate phases.

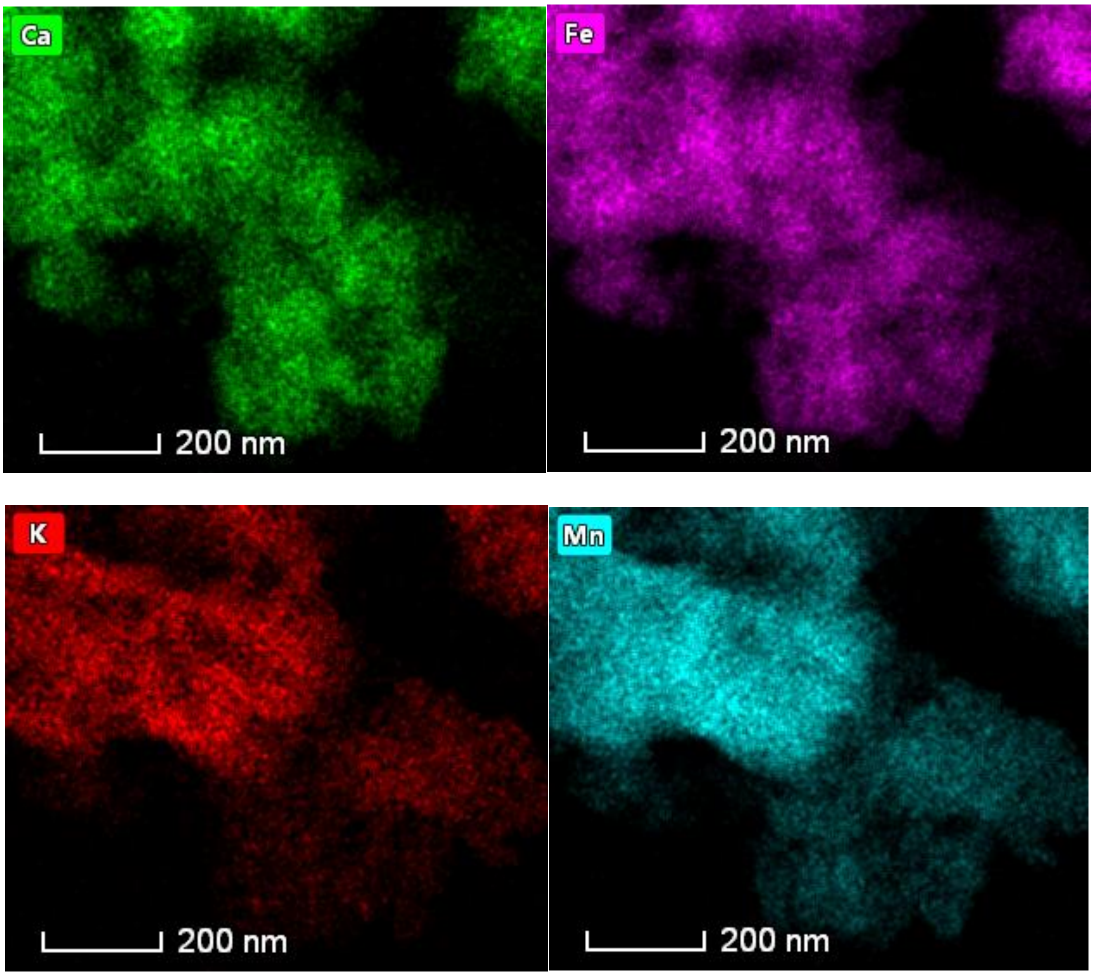

Supplementary Fig. 5 elemental and morphological analysis. TEM-EDX of fully oxidized $(Ca/Mn)_{1-x}O@KFeO_2$ redox catalyst.

For the fitting of Fe $2p_{3/2}$, we adopted the method by Grosvenor et al,[2] and the results are shown in Supplementary Fig. 6 (a-1) to (a-4). 4 multiplet peaks were used to fit for Fe^{3+}, and 3 multiplet peaks were used to fit for Fe^{2+}. It was determined that, oxidized and partially reduced redox oxide contains average Fe^{3+} on the surface, where deeply reduced redox catalyst contains average Fe^{2+} on the surface. For the fitting of Mn $2p_{3/2}$, we adopted the methods by Ilton et al. and Biesinger et al,[3,4] and used 4 multiplet peaks to fit for Mn^{4+}. As shown in Supplementary Fig. 6 (b-1) to (b-4), oxidized redox catalyst was mainly composed of Mn^{4+}, whereas reduced and deeply-reduced redox catalysts composed much larger Mn^{3+} and Mn^{2+} components.

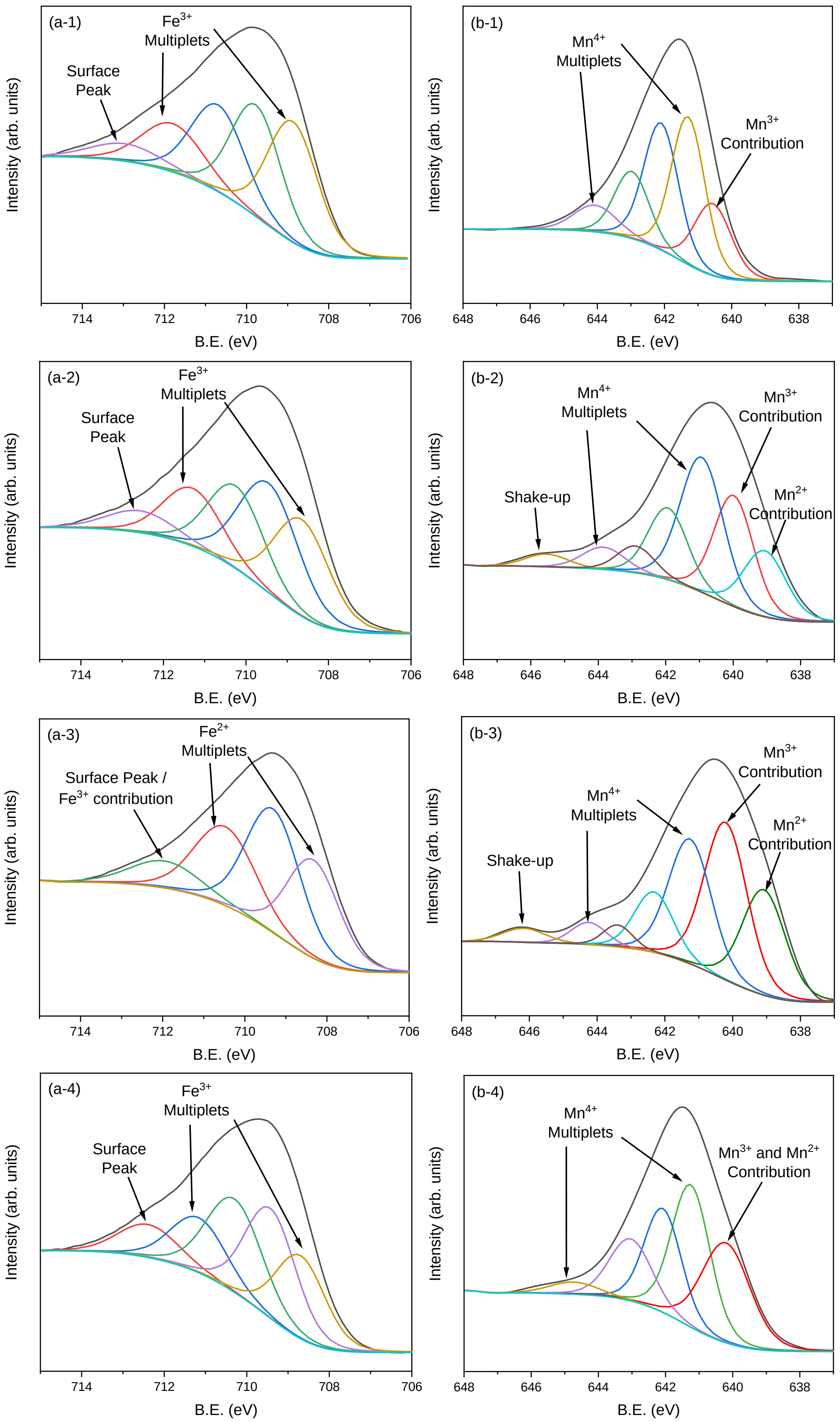

(a-1)
Fe3+ Multiplets
Surface Peak
Intensity (arb. units)
B.E. (eV)
(b-1)
Mn4+ Multiplets
Mn3+ Contribution
(a-2)
(b-2)
Shake-up
Mn2+ Contribution
(a-3)
Fe2+ Multiplets
Surface Peak / Fe3+ contribution
(b-3)
(a-4)
(b-4)
Mn3+ and Mn2+ Contribution

Supplementary Fig. 6 XPS analysis. (**a-1**) to (**a-4**): XPS Fe $2p_{3/2}$ peak fittings of oxidized, partially reduced, deeply reduced and long-term cycled (ending in oxidation step) $(Ca/Mn)_{1-x}O$@$KFeO_2$ redox catalysts; (**b-1**) to (**b-4**): XPS Mn $2p_{3/2}$ peak fittings of oxidized, partially reduced, deeply reduced and long-term cycled (ending in oxidation step) $(Ca/Mn)_{1-x}O$@$KFeO_2$ redox catalysts.

The Fe oxidation state of oxidized $(Ca/Mn)_{1-x}O$@$KFeO_2$ (Supplementary Fig. 7a) and partially $(Ca/Mn)_{1-x}O$@$KFeO_2$ (Supplementary Fig. 7b) were determined using Mössbauer spectroscopy. The Mössbauer spectroscopy of both oxidized and partially reduced $KFeO_2$@$CaMnO_{2+x}$ consist of three peaks. They are peaks from $KFeO_2$, 4-coordinated Fe-O_4 center in $Ca_2Fe_2O_5$ and 6-coordinated Fe-O_6 center in $Ca_2Fe_2O_5$. These peaks are almost consistent with each other in both oxidized and partially reduced $(Ca/Mn)_{1-x}O$@$KFeO_2$. As Mössbauer spectroscopy is a "bulk" technique, this reflects the average bulk oxidation of Fe is unchanged with partial reduction $(Ca/Mn)_{1-x}O$@$KFeO_2$ in the ethylbenzene ODH process (within the regular operating range).

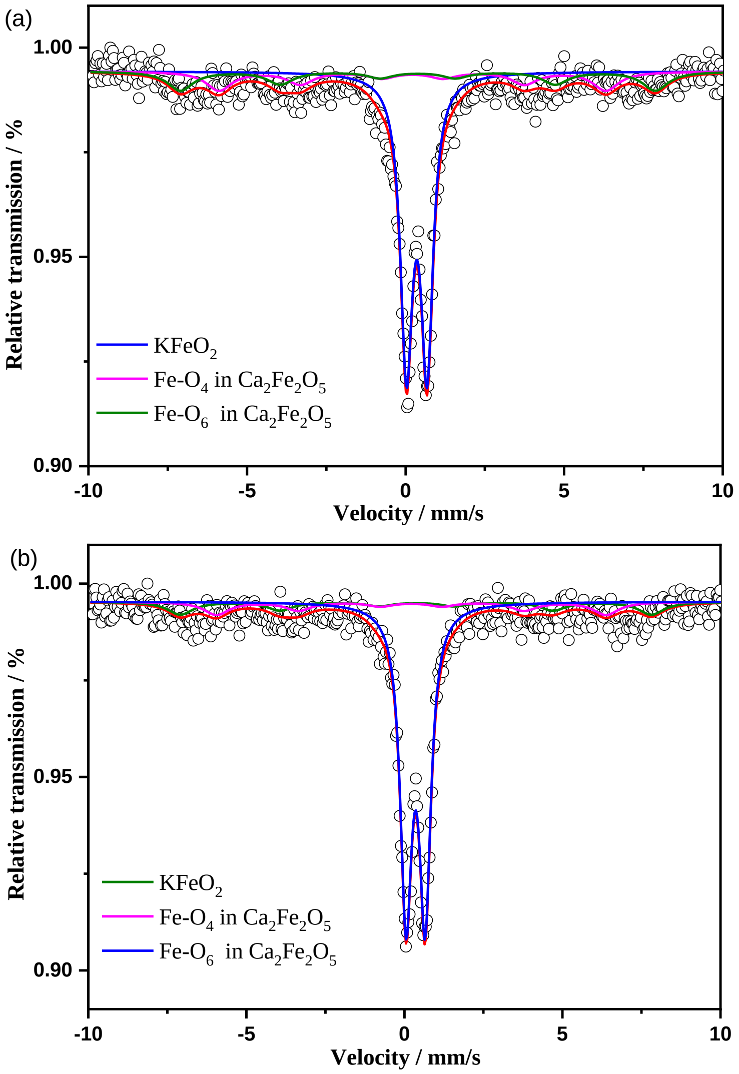

Supplementary Fig. 7 Mössbauer analysis. *Ex-situ* Mössbauer results on fully oxidized (**a**) and partially reduced (**b**) $(Ca/Mn)_{1-x}O$@$KFeO_2$ under operating scheme.

Based on literatures, the multiplet peaks of O *1s* peak can be assigned to lattice oxygen peak, carbonate oxygen peak and hydroxyl oxygen peak.[5] It was determined in Supplementary Fig. 8 that oxidized redox catalyst was comprised with a large fraction of lattice oxygen peak (64%) and a small fraction of carbonate oxygen peak (22%), whereas partially reduced redox catalyst was composed of a much smaller lattice oxygen peak (17%) and a much larger carbonate oxygen peak (80%). This result is consistent with the in-situ FTIR results in Fig. 5, where surface carbonate was formed upon injecting ethylbenzene onto the redox catalyst.

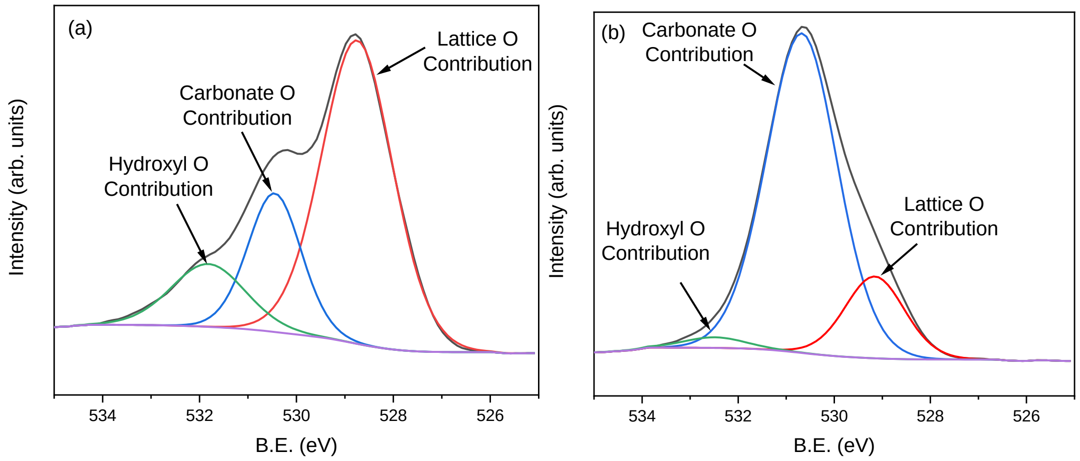

Supplementary Fig. 8 Oxygen species analysis. XPS O *1s* peak fitting of (**a**) oxidized $(Ca/Mn)_{1-x}O@KFeO_2$ redox catalyst and (**b**) partially reduced $(Ca/Mn)_{1-x}O@KFeO_2$ redox catalyst.

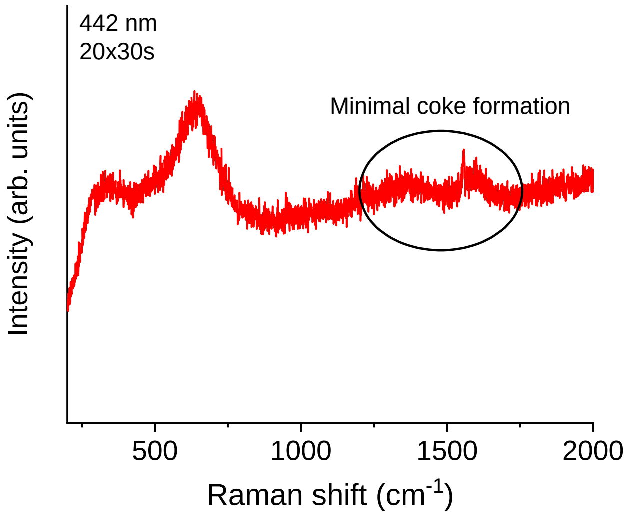

Supplementary Fig. 9 Raman spectroscopy analysis. Raman spectroscopy on used redox catalyst after ethylbenzene ODH for 10 mins.

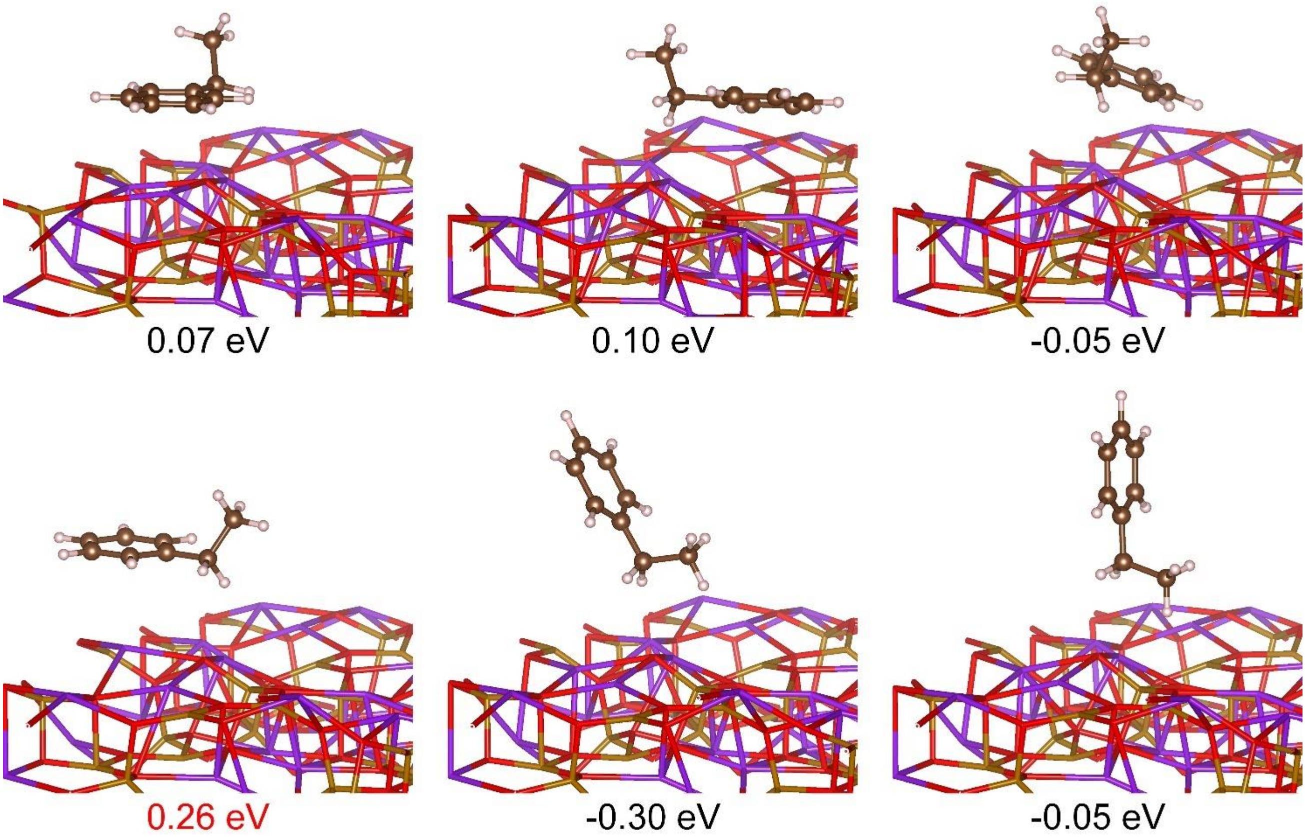

Supplementary Fig. 10 DFT caculation. Computed adsorption configurations for ethylbenzene to different sites of $KFeO_2$ (124) surface along various orientations together with corresponding adsorption energies in eV. The most stable one (red) was chosen for subsequent calculations.

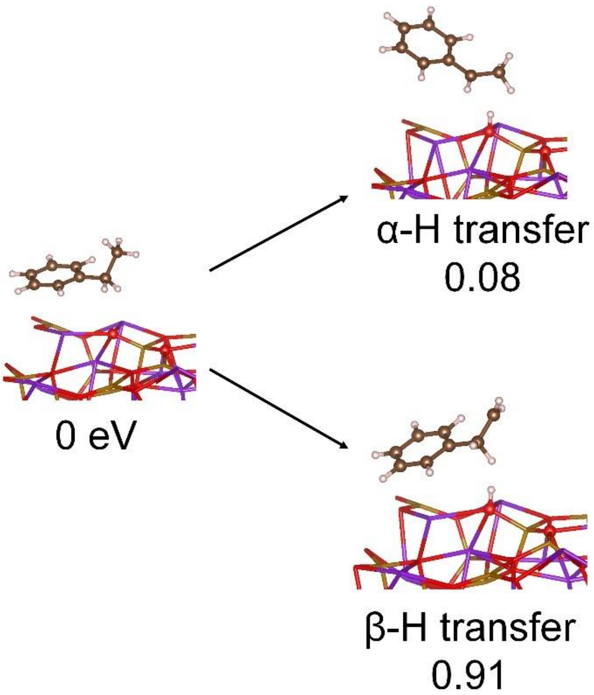

Supplementary Fig. 11 Energies of H transfer. Computed relative reaction energies of α- and β-H transfer

for ethylbenzene-ODH.

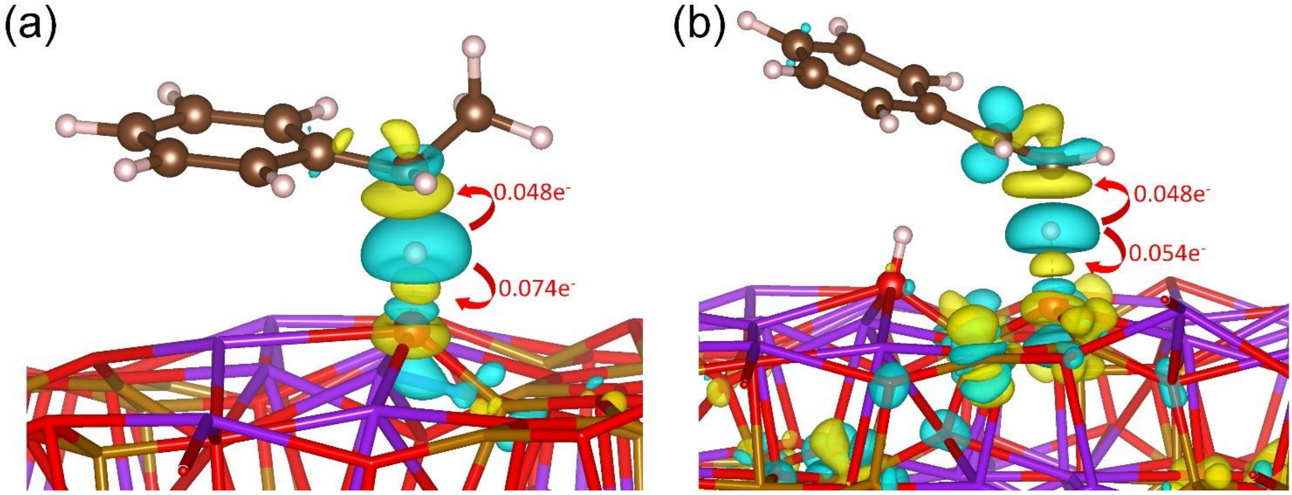

Supplementary Fig. 12 α and (b) β hydrogen abstraction. Computed electron density shifts in the transition state structures of (a) α and (b) β hydrogen abstraction. Yellow and cyan areas represent electron density increase and decrease, respectively. The isosurface of electron density is set to 0.003 electron $Å^{-3}$.

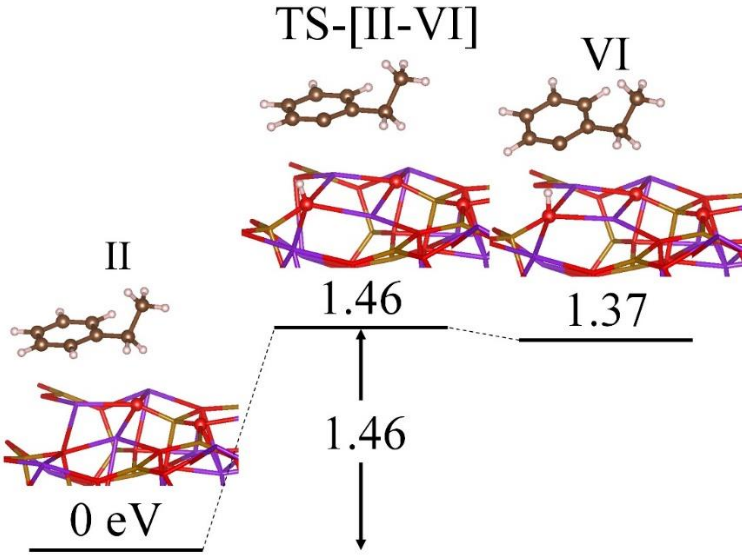

Supplementary Fig. 13 Energy potenial of H dissociation. Computed energy potential profiles of each elementary step for H dissociation on benzene ring in ethylbenzene

H-D exchange experiments at low temperatures (< 200 ºC) were conducted with TPSR-FTIR by absorbing D-ethylbenzene onto H-hydroxylated redox catalyst. The H-hydroxylated redox catalyst was prepared by treating the redox catalyst with H_2/O_2 redox cycles under 600 ºC for two cycles. And then the redox catalyst was cooled down to 100 ºC. It was observed that ethylbenzene is activated even at as low as 100 ºC, as indicated by the exchanged C-H peak bands observed between 2600 and 3000 cm^{-1} (Supplementary Fig. 14). It is noted that only exchanged bands assigned for ethyl branch C-H were observed, whereas C-H bands assigned for the benzene ring are absent. This indicates that only C-H at the ethyl branch are activated at lower

temperatures. On the contrary, another TPSR-FTIR experiment was done by absorbing ethylbenzene onto D-hydroxylated redox catalyst. The D-hydroxylated redox catalyst was prepared by treating the redox catalyst with D_2/O_2 redox cycles under 600 ºC for two cycles and then cooled to 100 ºC. Minimal peaks assigned to C-H could be detected. This is likely to be due to the limited dissociation rate of C-D due to kinetic isotope effects as described in the main text (Fig. 6b).

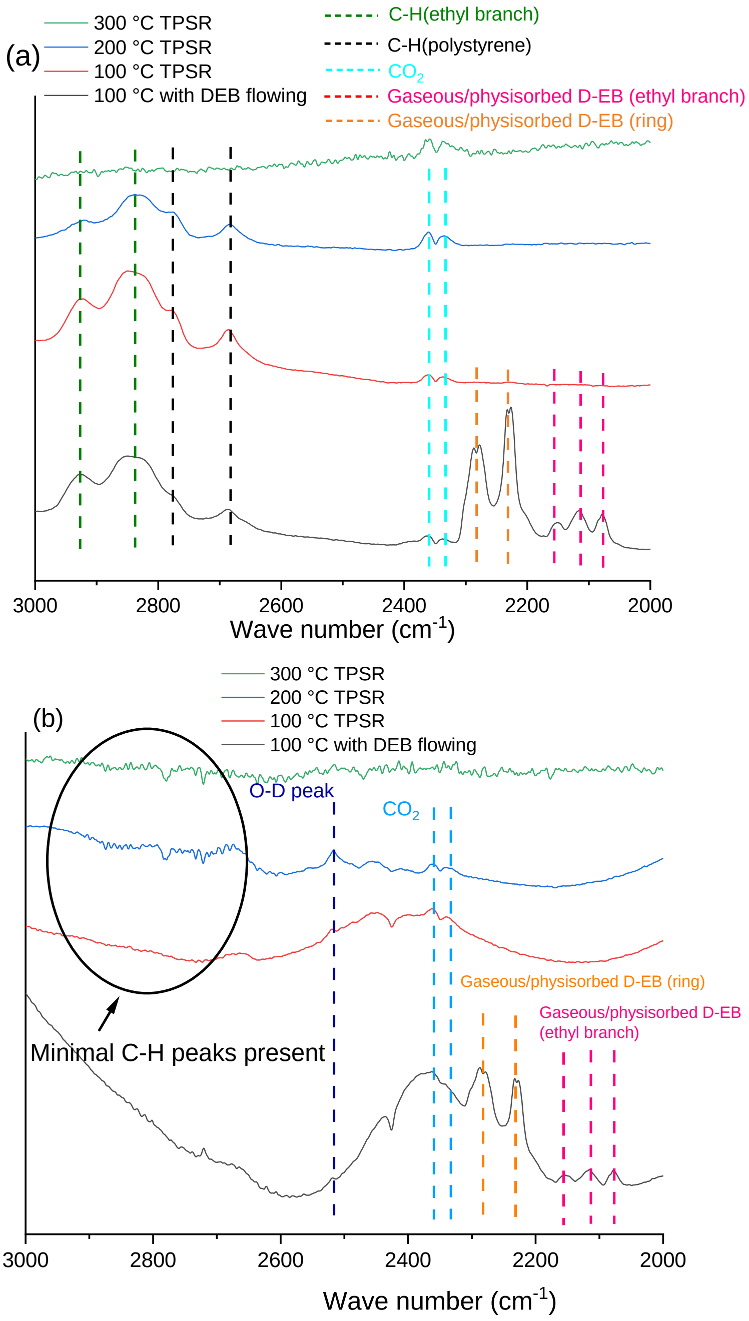

Supplementary Fig. 14 TPSR-FTIR. TPSR-FTIR of (**a**) D-ethylbenzene adsorption on H-hydroxylated $(Ca/Mn)_{1-x}O@KFeO_2$ and (**b**) ethylbenzene adsorption on D-hydroxylated $(Ca/Mn)_{1-x}O@KFeO_2$.

To prove the importance of water cofeed and the role of $(Ca/Mn)_{1-x}O$, ethylbenzene Redox-ODH was conducted on standalone $KFeO_2$ without water cofeed. As shown in Supplementary Fig. 15 for the on-line product distributions, styrene formation quickly decayed, indicating that ethylbenzene conversion decreased quickly. The overall ethylbenzene conversion (44.3%) was much lower than that on $(Ca/Mn)_{1-x}O@KFeO_2$.

Additionally, there is significant amount of H_2 signal detected by the QMS, indicating that the H_2 by-product was not effectively oxidized to H_2O. These results further confirmed that $(Ca/Mn)_{1-x}O$ is important in oxidizing the H_2 by-product to H_2O and to increase ethylbenzene conversion.

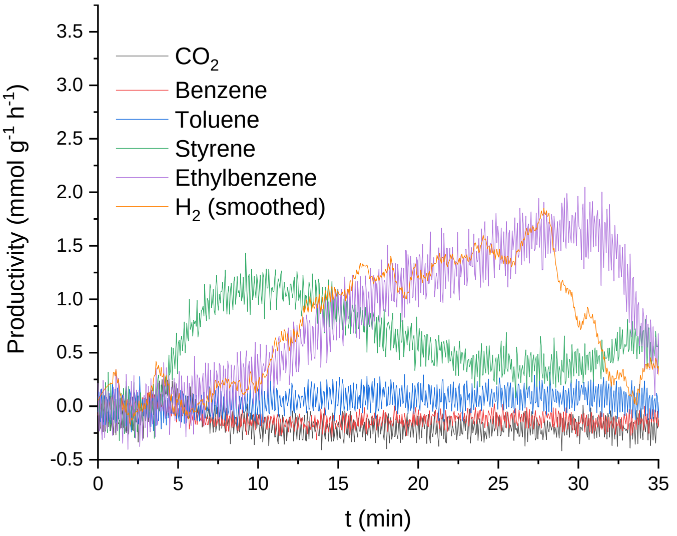

Supplementary Fig. 15 Product distributions. On-line product distributions of ethylbenzene Redox-ODH on standalone $KFeO_2$

Aspen Plus simulation:

Three cases have been analyzed to explore the impact of increased EB-to-styrene yield on the overall process energy savings.

- **Case 1**: Industrial catalytic dehydrogenation of EB to styrene
- **Case 2**: Redox-ODH of EB to styrene (10% EB in steam)
- **Case 3**: Redox-ODH of EB to styrene (product yields as in **Case 2**; no steam addition)

All the cases have been simulated using AspenPlus™ software. The product distribution for the three cases are given below:

Supplementary Table 1: Product distribution for **Case 1** [6], **Case 2** and **Case 3** (experimental)

	**Case 1**
**EB conversion**	**64%**
Product distribution (mol.%, dry basis)	
EB	16.5
Styrene	24.7
Benzene	3.8
Toluene	3.5
H_2	42.3
C_2H_4	2.1
CH_4	0.3
CO	1.7
CO_2	4.8
C	0.0

	**Case 2/Case 3**
**EB conversion**	**97%**
Product selectivity (C basis, %)	
EB	-
Styrene	94.2
Benzene	1.1
Toluene	2.3
H_2	-
C_2H_4	-
CH_4	0.3
CO	-
CO_2	0.2
C	1.9

***Case 1**: Industrial catalytic dehydrogenation of EB to styrene*

Catalytic dehydrogenation of ethylbenzene is the major industrial route for styrene production[7]. The endothermic reaction is carried out in the vapor phase with steam, over a catalyst consisting primarily of iron oxide. Based on the existing literature [6–9], a series of two adiabatic reactors is assumed for EB conversion, as shown in Supplementary Fig. 16. The feed to each reactor is in 650-675°C. Since the adiabatic reaction drops the temperature after the first reactor, the stream is heated back up to 650°C before entering the second reactor. EB concentration to first reactor is maintained at 5% at the inlet with the remaining primarily being steam. The feed also contains the recycled unreacted EB stream. Dilution steam prevents formation of coke from EB.

A single-pass EB conversion of 40% is assumed in each reactor, making the combined single-pass conversion to be 64%. An operating pressure of 2 bar is assumed [6,8,9]. The reactor effluent is fed through a heat recovery system to minimize energy consumption, condensed, and separated into vent gas, a crude styrene hydrocarbon stream, and a steam condensate stream. The crude styrene goes to a distillation system to separate into 99.9% pure styrene and unreacted EB, which is recycled. Ethylbenzene and styrene, having similar boiling points, require 70 – 100 trays for their separation. The 82-stage column operates under vacuum with a reflux-drum pressure of 10 kPa to give pure styrene. The second column for recycle EB has 38 stages and operates slightly above atmospheric pressure. Separation scheme also produces an aromatic stream rich in benzene and toluene, along with vent streams containing mainly CO_2 and H_2.

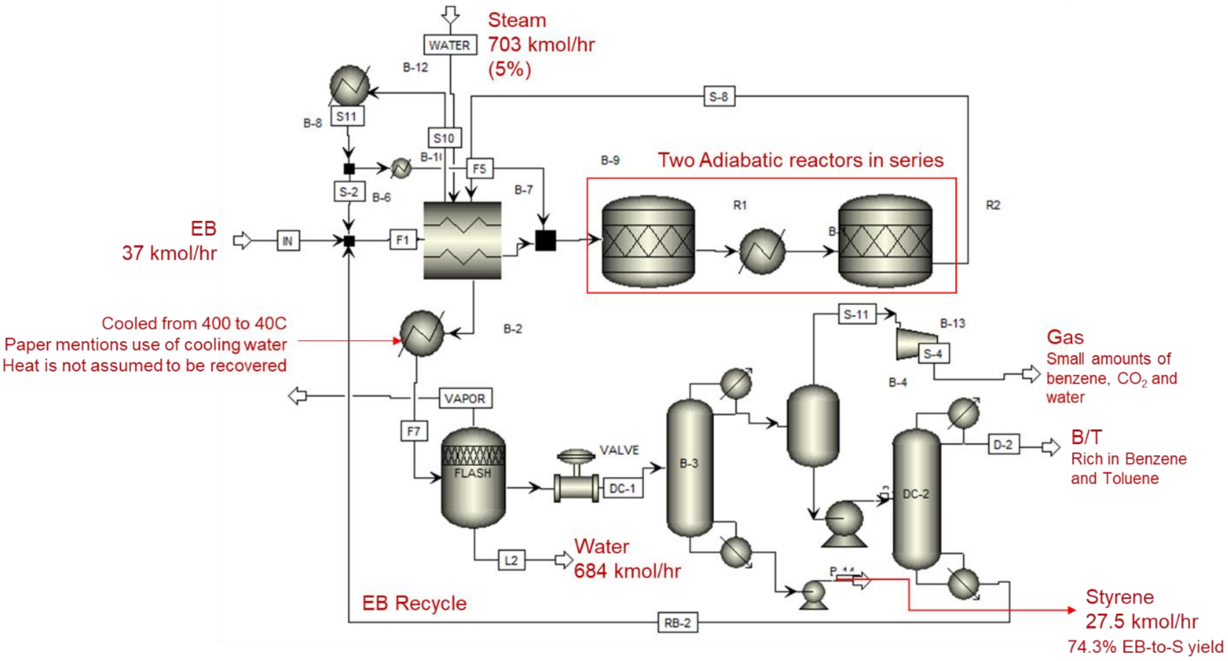

Supplementary Fig. 16 Case 1 process flow diagram. AspenPlus process flow diagram for **Case 1** (Industrial EB to styrene)

Supplementary Table 2: Reactions considered for modeling the **Case 1** EB dehydrogenation reactor based on [6]

Ethyl benzene → Styrene + H_2
Ethyl benzene → Benzene + C_2H_4
Ethyl benzene + H_2 → Toluene + CH_4
0.5 C_2H_4 + H_2O → CO + 2 H_2
CH_4 + H_2O → CO + 3 H_2
CO + H_2O → CO_2 + H_2

Toluene + H_2 → Benzene + CH_4
Styrene + H_2 → Benzene + C_2H_4

***Case 2**: Redox-ODH of EB to styrene (10% EB in steam)*

Supplementary Fig. 17 shows the reaction and separation scheme for Redox-ODH of EB to styrene. MnO_2↔MnO transition is used in the model, to mimic the change in Mn-oxidation state in the original redox material. Product distribution is listed in **Supplementary Table 1** above. To accurately reproduce the single pass yield and heat of reactions based on experimental results, the reducer reactor was modeled in ASPEN with two RStoic reactors. The first RStoic step converts EB whereas the second step combusts hydrogen with MnO_2 (redox catalyst). 100% H_2-to-H_2O combustion is assumed, based on experimental results. EB: steam ratio entering the first reactor is 1:9. The two reactors in series (reducer) together are adiabatic, first one being endothermic and the second exothermic. The reduced MnO is oxidized in a following reactor (oxidizer) using 20% excess air. The regenerated MnO_2 is recycled back to the reducer reactors. Hot, O_2-deficient air is utilized in generating the feed steam. The downstream separation scheme is kept similar to **Case 1**, to perform a consistent basis for comparison.

***Case 3**: Redox-ODH of EB to styrene (product yields as in **Case 2**; no steam addition)*

Case 3 represents a case where no steam addition is required **(**Supplementary Fig. 18). Here, EB conversion and product yields are as used in **Case 2** (**Supplementary Table 1**). The rest of the system is as described in **Case 2**.

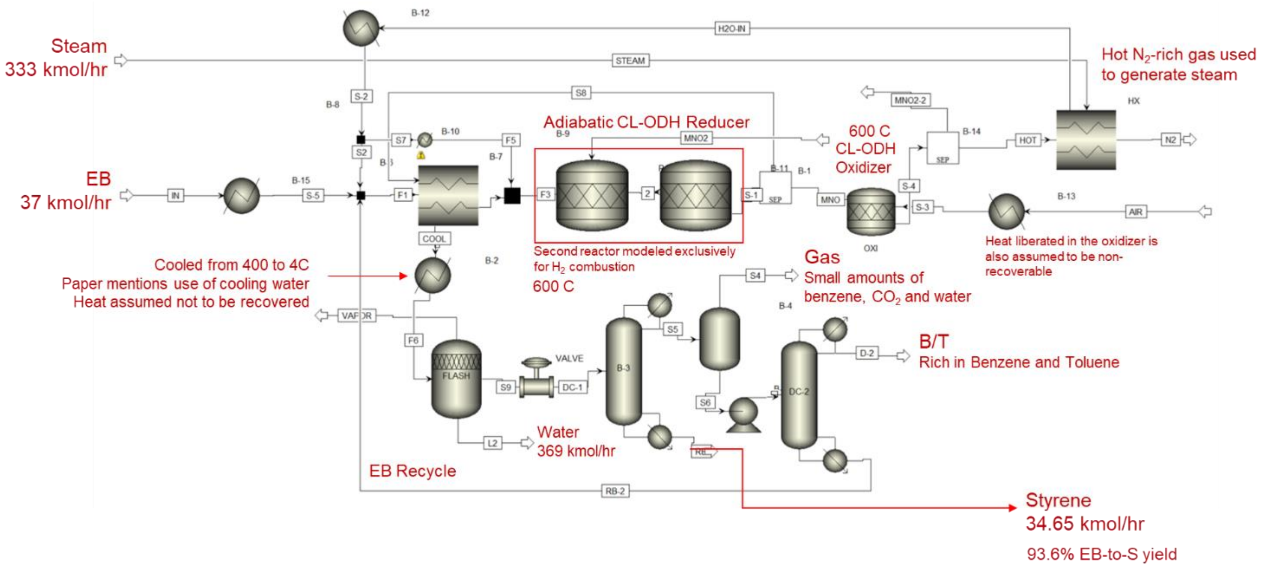

Supplementary Fig. 17 Case 2 process flow diagram. AspenPlus process flow diagram for **Case 2** (Redox-ODH of EB to styrene)

Supplementary Table 3: Reactions used to model the Redox ODH reactors for **Case 2** and **Case 3**

Redox ODH Reducer
Ethyl benzene → Styrene + H_2
6. Ethyl benzene → 8. Benzene + 6. H_2
Ethyl benzene + H_2 → Toluene + CH_4
Ethyl benzene → 8. C(s) + 5. H_2
2. Ethyl benzene + 42. MnO_2 (s) → 16. CO_2 + 42. MnO (s) + 10. H_2O
MnO_2 (s) + H_2 → MnO (s) + H_2O

Oxidizer
$MnO\ (s) + O_2 \rightarrow MnO_2\ (s)$

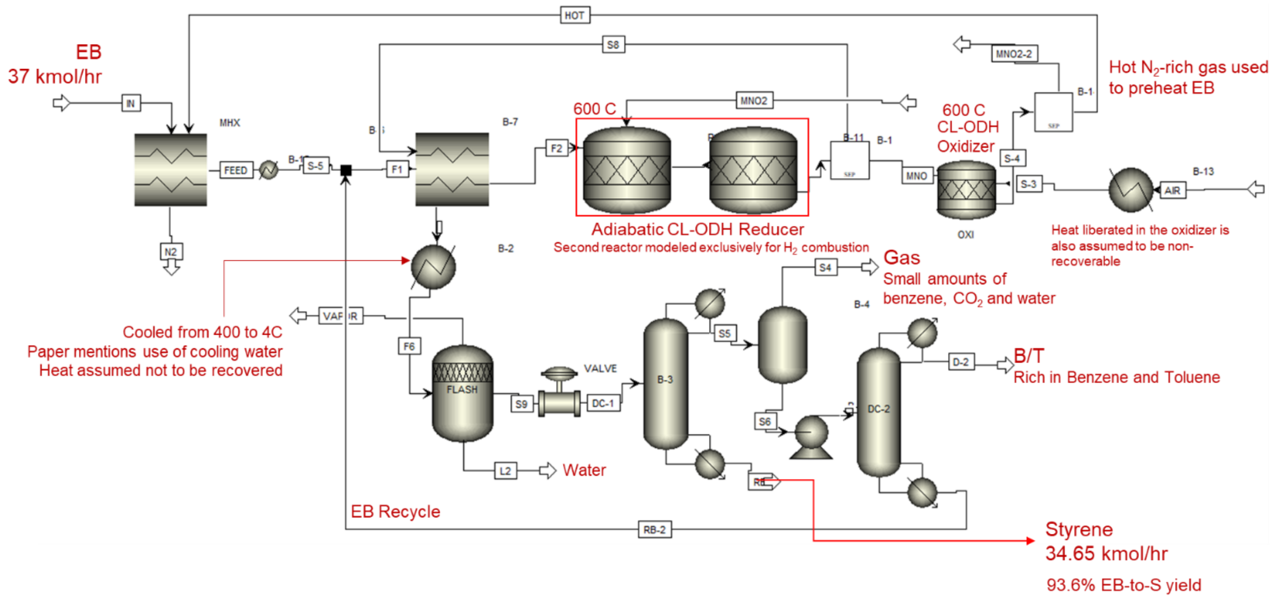

Supplementary Fig. 18 Case 3 process flow diagram. AspenPlus process flow diagram for **Case 3** (Redox-ODH of EB to styrene)

The various methods and assumption used in the simulations are listed in **Supplementary Table 4** below:

Stream class	MIXCISLD
Databank	PURE, AQUEOUS, SOLIDS, INORGANIC, AP EOS
Solid components	MnO_2, MnO and C
Property method	PETCHEM filter with PENG-ROB method and STEAM –TA for steam cycles
Unit operation models	
**Case 1**: Adiabatic EB reactors **Case 2 and Case 3**: Redox ODH reactors (both reducer and oxidizer)	RStoic
Pressure changers	Pump
Heat exchangers	Heater and MHeatX
Distillation columns	DSTWU
Separators/Flash columns	Sep/Flash2 and Flash 3 (for 3 phase)

Assumptions	
Basis	100 tonne/day of feed EB
Ambient condition	T = 25°C, P = 1 atm
Redox ODH Reducer	Operating pressure: 1 atm
	MnO_2: 20% excess, compared to stoichiometric requirement
Redox ODH Oxidizer	20% excess air
	100% H_2-to-H_2O conversion
	Temperature: 600°C, Exothermic heat from oxidizer not recovered

Product cooling	Cooling water is used. Heat not recovered. Low quality
Distillation columns	Condenser duties not included in calculating overall process heat duty (**Supplementary Table 5** and Supplementary Fig. 18)
MHeatX Temperature approach	10 K
Thermal energy conversion efficiency	To steam: 85%
	To electric energy: 40%

Optimization strategy

Case 1 is simulated based on the process described in literature. For **Case 2** and **Case 3**, certain upstream parameters are modified to optimize the process to compare with **Case 1**.

Objective: To operate the two reactor system for Redox ODH reducer adiabatically, keeping the reactor outlet at 600°C.

Constants: Product distribution (**Case 2** and **Case 3**, as listed in **Supplementary Table 1**), steam input, EB input and the amount of redox catalyst.

Variables

- Amount of steam mixed with feed EB and recycle, before the stream has exchanged heat with the reactor outlet stream (X_s)
- Temperature of the stream (fresh EB + Xs amount of steam + recycle) before mixing with the remaining steam
- Temperature of the oxidizer (temperature of the reduced solids entering the reducer) [(600 ± 50)° C]

Both the reducer reactors in **Case 2** and **Case 3** are operated at 600°C. The above variables are used to ensure the total Q of both the reactors equals zero (±0.5%).

The optimized conditions result in all of the steam being mixed with EB (fresh and recycled) before heat exchange, with the output of the stream at 590°C. The oxidizer temperature is set at 630°C, which allows for adiabatic operation of the two reactors, with an outlet temperature of 600°C.

Aspen Plus Simulation Results:

Supplementary Table 5 lists the heat duty required for the different sections of the process, for all three cases. The values are normalized to per tonne of styrene produced. These results are obtained based on the assumptions in **Supplementary Table 4** and models developed in Aspen Plus. **Case 1** yields an overall heat duty demand of 16.7 GJ/tonne styrene, which is in close agreement with the value reported in literature [10]. Reports in literature show various optimized process plant configurations for **Case 1**, but for this study, the most widely used base case is adopted as **Case 1**. Redox ODH **Case 2** depicts the experimental results on the redox catalyst which is the focus of this study. This provides an overall styrene yield of 91.4%, which is 38.4% higher than the reported value for **Case 1** (53%).

Case 2 leads to a decrease in the heat duty requirement by roughly 50% compared to **Case 1**. Major savings arise from the substantial drop in steam requirement for Redox ODH schemes, which can operate with low coke and high styrene selectivity at 0.1 atm EB partial pressure. In our experimental studies, we used 0.1 atm EB balance inert Ar. **Case 2** conservatively assumed that a 1:9 EB to steam molar ratio is used in redox-ODH (steam is used in the place of Ar). This results in >50% reduction in steam usage compared to the

commercial DH process. **Case 3** covers a more optimistic case, which assumes that “dry” EB can be used without steam to achieve product yields and selectivity identical to those obtained from our experimental redox-ODH unit with 0.1 atm EB balance Ar. We note that our experimentally obtained EB conversion and styrene yield are significantly higher than those in conventional DH. This increased EB yield also contributes to lower energy requirements in the EB separation column. Comparison of the styrene separation columns in **Case 1** and **Cases 2/3** are shown in **Supplementary Table 6**. Overall, the redox ODH process, as analyzed in **Cases 2/3**, allows for highly efficient styrene production from EB, reaching 91.4% yields, with a heat duty demand half of what the industrial EB dehydrogenation route demands. Redox ODH process can operate at high EB: steam ratios, leading to significant savings in steam requirement, which is the prime source of energy demand.

Supplementary Table 5: Section-wise heat duty requirement for the different cases

**Process Section**		**Case 1**	**Case 2**	**Case 3**
		Heat duty (GJ/tonne styrene)		
EB feed preheat		0	0.75	0
Steam generation		7.01	4.45	0
Steam preheating		5.29	0	0
Interstage product heating		0.62	0	0
Styrene separation column	*Condenser**	*-2.81*	*-2.86*	*-2.84*
	Reboiler	3.39	3.06	3.04
EB separation column	*Condenser**	*-0.28*	*-0.03*	*-0.03*
	Reboiler	0.44	0.05	0.05
Reactor 1		0		
Reactor 2		0		
ODH Reducer			0	0
Air preheat			0	0
Regenerator			-1.29	-1.28
Product cooling#		-11.1		
**Total**		**16.7**	**8.3**	**3.1**

* Condenser duties (heat removal) are satisfied by cooling water which is not counted towards the overall energy requirements;

Heat released from product cooling is of very low grade (low pressure steam condensation) and is therefore not recoverable

Supplementary Table 6: Comparison of the styrene separation column in **Case 1** and **Case2/Case3**

**Column parameter**	**Case 1**	**Case 2/Case 3**
Styrene purity (vol./ mol%)	99.9%	99.9%
Condenser pressure	0.1	0.1
Reboiler pressure	0.5	0.5
Minimum reflux ratio	5.9	43.9
Actual reflux ratio	7.1	53.0
Minimum number of stages	39.3	29.3
Number of actual stages	82.0	61
Feed stage	30.7	32.2

Number of actual stages above feed	29.7	31.2
Distillate temperature	59°C	47°C
Bottom temperature	121°C	121°C
Distillate to feed ratio	0.51	0.13

Cartesian coordinates of the key TS structures in the format of VASP output.

1. TS-[I-II]

```
1.00000000000000
  16.1327991486000002    0.0000000000000000    0.0000000000000000
  -0.0242462366000000   22.8120865715000001    0.0000000000000000
   0.0000000000000000    0.0000000000000000   20.4906997681000007
Fe   K   O   C   H
32   32  64  8   10
Selective dynamics
Direct
  0.3120000059999978  0.5663300160000020  0.0711499969999991   F   F   F
  0.5466285139408117  0.7087424548893630  0.2035223441903394   T   T   T
  0.9340482507861719  0.0571274338291047  0.1590039334654700   T   T   T
  0.6887500289999977  0.9335399870000032  0.0576500000000024   F   F   F
  0.0673923272704888  0.1739335963183907  0.1590008874321842   T   T   T
  0.8137500289999977  0.0563700009999977  0.0600599979999998   F   F   F
  0.1870000059999981  0.4435000120000012  0.0687400030000020   F   F   F
  0.4391078986593071  0.5736166302527526  0.1911452810367640   T   T   T
  0.6779199839999990  0.4389500019999986  0.0636800010000016   F   F   F
  0.9358583934440590  0.5640832235935960  0.1881863899915033   T   T   T
  0.3228299920000139  0.0609200000000030  0.0651199969999965   F   F   F
  0.5696134468267413  0.1963083737291894  0.1856676655879566   T   T   T
  0.4488368657169085  0.0677524784431778  0.1808054109239516   T   T   T
  0.1978400050000033  0.9380800129999969  0.0627100020000029   F   F   F
  0.0603777478388134  0.6881958294198880  0.1700175075715228   T   T   T
  0.8029099699999946  0.5617799759999969  0.0660900029999993   F   F   F
  0.1865600049999969  0.6926500200000021  0.0732799990000004   F   F   F
  0.4327604628113034  0.8301737732709126  0.1992969956686333   T   T   T
  0.0589677026614683  0.9327641236539792  0.1760614057583759   T   T   T
  0.8141899700000010  0.8072100280000002  0.0555200020000015   F   F   F
  0.1891900000000035  0.1794600039999992  0.0585899989999987   F   F   F
  0.4414930993513893  0.3178648402489691  0.1909305895702708   T   T   T
  0.0599759620530105  0.4355370372596321  0.1979682158583163   T   T   T
  0.8115599750000015  0.3204100130000000  0.0702100020000032   F   F   F
  0.2996900080000105  0.3144600090000012  0.0635899979999976   F   F   F
  0.5604242874839660  0.4518982512559654  0.1642611378722419   T   T   T
  0.7010599970000015  0.1853999940000008  0.0652100000000004   F   F   F
  0.9542400403795255  0.3120700207852635  0.1931326193384902   T   T   T
  0.3260700109999988  0.8131600020000036  0.0621399990000029   F   F   F
```

0.5704922351017990	0.9464969017300118	0.1952837354191454	T	T	T
0.6746799949999982	0.6867099999999979	0.0666600019999990	F	F	F
0.9311525069454971	0.8089876320450363	0.1789577374207247	T	T	T
0.4349099989999985	0.6928799749999968	0.0672200019999991	F	F	F
0.6765517617014800	0.8255374529538333	0.2192565743871368	T	T	T
0.5658400060000019	0.8069900269999977	0.0615799990000028	F	F	F
0.8153971761978753	0.9420181265135911	0.2007770418081519	T	T	T
0.1707926698251730	0.2881065120615008	0.2381559949482851	T	T	T
0.9408500190000102	0.1849000010000026	0.0583400020000013	F	F	F
0.0599000010000026	0.3149699869999978	0.0704599990000006	F	F	F
0.3141870153596635	0.4316537504307784	0.2105179700843024	T	T	T
0.5548899770000020	0.3124600049999984	0.0676700030000035	F	F	F
0.8218169202293613	0.4477775797456996	0.2265795516324279	T	T	T
0.4458599980000060	0.1874099970000032	0.0611299979999984	F	F	F
0.6862914221884332	0.3295985340874316	0.1966363061354850	T	T	T
0.0708499999999996	0.8094900250000023	0.0643699990000002	F	F	F
0.3164952142145709	0.9419165421533926	0.2200634776505005	T	T	T
0.1695760346182719	0.8206197112191120	0.2300586940795263	T	T	T
0.9298999909999992	0.6903700229999998	0.0644299980000014	F	F	F
0.0640299989999994	0.5736399890000001	0.0724299999999971	F	F	F
0.3178484731662894	0.7094072219206996	0.2235038963910407	T	T	T
0.1830403842570131	0.0596727789854615	0.1909412654143158	T	T	T
0.9367200140000023	0.9262200000000007	0.0563700009999977	F	F	F
0.0617300010000008	0.0552100020000026	0.0519200000000026	F	F	F
0.3308076934971239	0.1938255271574164	0.1927185166680715	T	T	T
0.1885586420756599	0.5698238615509428	0.2055338053806207	T	T	T
0.9390199780000046	0.4446499940000024	0.0768800009999993	F	F	F
0.4194500150000025	0.4348599910000033	0.0659700040000004	F	F	F
0.6788800647724575	0.5679041096594137	0.2075224836314445	T	T	T
0.5813000200000005	0.0650099960000006	0.0628300010000018	F	F	F
0.8305660945490402	0.1924753368205616	0.1925210205882862	T	T	T
0.6838783294755107	0.0673024046651785	0.2293735806278388	T	T	T
0.4562999899999980	0.9360200169999970	0.0672700029999973	F	F	F
0.5444599989999972	0.5638499860000010	0.0615300010000013	F	F	F
0.8098616499541823	0.6874656669304404	0.1988194977871802	T	T	T
0.2601228326886724	0.7509628260254674	0.0449007756350741	T	T	T
0.4675073848825569	0.9090514447529893	0.2018797910782296	T	T	T
0.0235183600459585	0.8562380439908730	0.1875415715623697	T	T	T
0.7648800020000226	0.7331200240000015	0.0370399990000010	F	F	F
0.3989317805608074	0.2939469471493639	0.1017861769395139	T	T	T
0.6750224931747785	0.4456173019019413	0.1672816691489144	T	T	T
0.6109799740000028	0.2119999969999995	0.0135300000000029	F	F	F
0.8490897333279399	0.3333080578891773	0.1602772098675188	T	T	T
0.2089899930000030	0.2610599989999969	0.0681300010000001	F	F	F

0.4758636404473686	0.3963388178108785	0.1817346134545884	T	T	T
0.0444526239451839	0.3579253123814258	0.2218645449543505	T	T	T
0.7917600270000049	0.2388100029999975	0.0606700000000018	F	F	F
0.4249642316006192	0.8064425763122359	0.1051237313429191	T	T	T
0.6640838512796394	0.9408508217555260	0.2400689557055338	T	T	T
0.5840700270000028	0.6907699699999981	0.0071999999999974	F	F	F
0.8222297213887568	0.8208428254009887	0.1505570307985836	T	T	T
0.0738291299938583	0.6874807680563494	0.0722311106739723	T	T	T
0.3289363603767938	0.8226665139190218	0.2299546187551250	T	T	T
0.1711052729067375	0.9384570639084258	0.1574040849441820	T	T	T
0.9197999840000008	0.8067899939999990	0.0135999999999967	F	F	F
0.2947399910000001	0.1418000010000000	0.0581299999999985	F	F	F
0.5398778333159913	0.2750517038095244	0.1977454746539409	T	T	T
0.7060099839999978	0.3580600020000019	0.0706700010000034	F	F	F
0.9672580436204153	0.4848033238729072	0.1972677550599027	T	T	T
0.2748388867896404	0.3873029277710117	0.0919029462704613	T	T	T
0.5338652394923818	0.5263251933898588	0.1948289520168613	T	T	T
0.7391800279999998	0.1139999999999972	0.0271799989999977	F	F	F
0.9909631394133347	0.2347664661881254	0.1713076671667990	T	T	T
0.8231972130879627	0.0741152916059996	0.1656060049988791	T	T	T
0.5989752841453840	0.9161851023696461	0.1054424660168065	T	T	T
0.3866899909999972	0.5958499910000015	0.0069799999999987	F	F	F
0.6447949605029228	0.7082006221119818	0.1589461476139345	T	T	T
0.2295673182049224	0.6208897715978590	0.0937485347046964	T	T	T
0.5223354329234128	0.7817754383504709	0.2322537248956789	T	T	T
0.9649803812635340	0.9791906033965951	0.1735657762164476	T	T	T
0.7508699889999946	0.8702700139999991	0.0216899999999995	F	F	F
0.0808149162905497	0.1637783324372172	0.0616728460176608	T	T	T
0.3544398798625908	0.3070459969517406	0.2485765669485462	T	T	T
0.1499327139750246	0.4672524989286456	0.1525829111461006	T	T	T
0.8750200269999991	0.3507800100000011	0.0000000000000000	F	F	F
0.5656577235569781	0.4523661086734413	0.0664310585512781	T	T	T
0.8354244689310768	0.5739882031556588	0.2236470696409544	T	T	T
0.4184600110000005	0.0453499999999991	0.0133399999999995	F	F	F
0.6762585231809065	0.1760118314097499	0.1578367403259556	T	T	T
0.5464623142033682	0.0263330184689494	0.1830630706293630	T	T	T
0.2934199870000000	0.8915699719999993	0.0453899990000011	F	F	F
0.7160148804462665	0.6097471406401775	0.0854672905778858	T	T	T
0.9642320933079850	0.7312463729983770	0.1875065825798740	T	T	T
0.2467399980000044	0.5069199799999993	0.0306100009999994	F	F	F
0.4614389948362648	0.6566375290773782	0.1963997152137646	T	T	T
0.0245697061253051	0.1015278824241177	0.1862719797891291	T	T	T
0.7694260905553882	0.9837507544558163	0.0781561479543273	T	T	T
0.1793640763434862	0.1776077595417556	0.1700009237628894	T	T	T

0.9238625384141990	0.0658185457729258	0.0620806828153437	T	T	T
0.1217800009999978	0.4070599969999975	0.0026000000000010	F	F	F
0.3432708000260843	0.5391660434019336	0.1567081026192203	T	T	T
0.0342251044028450	0.6112675029600367	0.1944339113411016	T	T	T
0.7700406888338085	0.4875841356515316	0.0452513886369091	T	T	T
0.2270399929999991	0.0167599990000014	0.0415600019999971	F	F	F
0.4785893402496140	0.1452958031492005	0.1852561894201629	T	T	T
0.3416703953395621	0.0479511732590380	0.1595330789426485	T	T	T
0.1020800020000010	0.9169099929999973	0.0135599999999982	F	F	F
0.1687038219806051	0.7156525959951783	0.1741607741451278	T	T	T
0.9102129152712035	0.5721700885443018	0.0914721365854929	T	T	T
0.2137934544643451	0.2226396485901539	0.3741969321693193	T	T	T
0.1278841412048613	0.2265594202797401	0.3806528702007444	T	T	T
0.0907118702949668	0.2800294550000262	0.3971533114636509	T	T	T
0.1412781314155743	0.3288884790939773	0.4087005917959363	T	T	T
0.2271753895203768	0.3248198383207022	0.4027874868382531	T	T	T
0.2662125371846081	0.2717798351694650	0.3839824384214553	T	T	T
0.3552083933074212	0.2682414344150292	0.3699944505014405	T	T	T
0.4149327984547442	0.3017680685481250	0.4135900939651998	T	T	T
0.2420901172786374	0.1804143792761513	0.3623121145822578	T	T	T
0.0895096012353560	0.1877162137696229	0.3727961958679462	T	T	T
0.0235113141210894	0.2835578769674283	0.4011326185533236	T	T	T
0.1131392647046623	0.3706367482353634	0.4223386407724686	T	T	T
0.2651219108577215	0.3634932628092197	0.4125243065866222	T	T	T
0.3592146920967118	0.2899075389966494	0.3073267645445684	T	T	T
0.3751241492985364	0.2224442743340967	0.3633764068106149	T	T	T
0.4000988137735310	0.3487170598432847	0.4170487032797484	T	T	T
0.4788115192171054	0.2983285368784128	0.3954101573379549	T	T	T
0.4143688002432880	0.2843505521951087	0.4639353321905684	T	T	T

2. TS-[II-III]

1.00000000000000

16.1327991486000002	0.0000000000000000	0.0000000000000000
-0.0242462366000000	22.8120865715000001	0.0000000000000000
0.0000000000000000	0.0000000000000000	20.4906997681000007

Fe	K	O	C	H
32	32	64	8	10

Selective dynamics

Direct

0.3120000059999978	0.5663300160000020	0.0711499969999991	F	F	F
0.5454569445486676	0.7094229284691427	0.2029476179237221	T	T	T
0.9326974458075672	0.0545590518425807	0.1596651443854207	T	T	T
0.6887500289999977	0.9335399870000032	0.0576500000000024	F	F	F
0.0661371979081070	0.1715197661071148	0.1572440814433460	T	T	T
0.8137500289999977	0.0563700009999977	0.0600599979999998	F	F	F

0.1870000059999981	0.4435000120000012	0.0687400030000020	F	F	F
0.4385889671420125	0.5744006171805999	0.1912275128574874	T	T	T
0.6779199839999990	0.4389500019999986	0.0636800010000016	F	F	F
0.9353871378983186	0.5648726177608571	0.1875624165216543	T	T	T
0.3228299920000139	0.0609200000000030	0.0651199969999965	F	F	F
0.5677120066078313	0.1959934281094417	0.1868214342542490	T	T	T
0.4484343892300261	0.0677413239644792	0.1811806582182347	T	T	T
0.1978400050000033	0.9380800129999969	0.0627100020000029	F	F	F
0.0609740639585750	0.6884874435938516	0.1702529886943878	T	T	T
0.8029099699999946	0.5617799759999969	0.0660900029999993	F	F	F
0.1865600049999969	0.6926500200000021	0.0732799990000004	F	F	F
0.4324827819680107	0.8299946624484265	0.2000548485178152	T	T	T
0.0578717662791689	0.9313429904211632	0.1772760607140150	T	T	T
0.8141899700000010	0.8072100280000002	0.0555200020000015	F	F	F
0.1891900000000035	0.1794600039999992	0.0585899989999987	F	F	F
0.4409554830028828	0.3187611487848720	0.1998097109246532	T	T	T
0.0588159459309364	0.4369758440696960	0.1981282038945565	T	T	T
0.8115599750000015	0.3204100130000000	0.0702100020000032	F	F	F
0.2996900080000105	0.3144600090000012	0.0635899979999976	F	F	F
0.5601542655162149	0.4519358904059442	0.1656139557964651	T	T	T
0.7010599970000015	0.1853999940000008	0.0652100000000004	F	F	F
0.9526256909313876	0.3117115816820642	0.1925842600466193	T	T	T
0.3260700109999988	0.8131600020000036	0.0621399990000029	F	F	F
0.5703008062163014	0.9460167024724987	0.1944636650018372	T	T	T
0.6746799949999982	0.6867099999999979	0.0666600019999990	F	F	F
0.9310101033723339	0.8081949122844638	0.1794508678043743	T	T	T
0.4349099989999985	0.6928799749999968	0.0672200019999991	F	F	F
0.6759260580150079	0.8247443712529255	0.2211452362153298	T	T	T
0.5658400060000019	0.8069900269999977	0.0615799990000028	F	F	F
0.8137175324696827	0.9394150403334627	0.2009470390218111	T	T	T
0.1630940337316077	0.2870746959224507	0.2159045293521619	T	T	T
0.9408500190000102	0.1849000010000026	0.0583400020000013	F	F	F
0.0599000010000026	0.3149699869999978	0.0704599990000006	F	F	F
0.3079341664851361	0.4340211520024559	0.2067269420215257	T	T	T
0.5548899770000020	0.3124600049999984	0.0676700030000035	F	F	F
0.8213106542941769	0.4487407830431092	0.2280042029086308	T	T	T
0.4458599980000060	0.1874099970000032	0.0611299979999984	F	F	F
0.6858741534308450	0.3296624098037549	0.1960393474646503	T	T	T
0.0708499999999996	0.8094900250000023	0.0643699990000002	F	F	F
0.3165227971277045	0.9417917533832481	0.2195719894348502	T	T	T
0.1704321951278148	0.8202012741410515	0.2293468241997817	T	T	T
0.9298999909999992	0.6903700229999998	0.0644299980000014	F	F	F
0.0640299989999994	0.5736399890000001	0.0724299999999971	F	F	F
0.3184426133574390	0.7099240796760170	0.2286251140365100	T	T	T

0.1828144598670476	0.0577715755481030	0.1907823492477378	T	T	T
0.9367200140000023	0.9262200000000007	0.0563700009999977	F	F	F
0.0617300010000008	0.0552100020000026	0.0519200000000026	F	F	F
0.3289315569396457	0.1934130801313055	0.1855273918194727	T	T	T
0.1885348045486376	0.5713156464392294	0.2052148325368921	T	T	T
0.9390199780000046	0.4446499940000024	0.0768800009999993	F	F	F
0.4194500150000025	0.4348599910000033	0.0659700040000004	F	F	F
0.6780766673556090	0.5689469492134996	0.2078000465906902	T	T	T
0.5813000200000005	0.0650099960000006	0.0628300010000018	F	F	F
0.8298241009280466	0.1926087802233959	0.1921230256931742	T	T	T
0.6835473186572580	0.0661492595793564	0.2283060766258155	T	T	T
0.4562999899999980	0.9360200169999970	0.0672700029999973	F	F	F
0.5444599989999972	0.5638499860000010	0.0615300010000013	F	F	F
0.8086974221591324	0.6881551015865236	0.1971152552141769	T	T	T
0.2598886045974103	0.7511601089352344	0.0448300739357825	T	T	T
0.4671777996636924	0.9087436626089928	0.2015324834801675	T	T	T
0.0239540146292211	0.8544390538108632	0.1884117910666113	T	T	T
0.7648800020000226	0.7331200240000015	0.0370399990000010	F	F	F
0.3977836909559496	0.2944001395698324	0.1029705439772089	T	T	T
0.6751234612065106	0.4463001370005927	0.1676030328262203	T	T	T
0.6109799740000028	0.2119999969999995	0.0135300000000029	F	F	F
0.8472520133689706	0.3325260932138984	0.1606012780609919	T	T	T
0.2089899930000030	0.2610599989999969	0.0681300010000001	F	F	F
0.4753427613994511	0.3965004987621231	0.1873055625114203	T	T	T
0.0398294673799784	0.3595675802485314	0.2222140587380738	T	T	T
0.7917600270000049	0.2388100029999975	0.0606700000000018	F	F	F
0.4240692091536362	0.8050440081900508	0.1063947231448242	T	T	T
0.6629977414104693	0.9395721268357831	0.2405022007183722	T	T	T
0.5840700270000028	0.6907699699999981	0.0071999999999974	F	F	F
0.8221892196701084	0.8197340625270698	0.1511018248084154	T	T	T
0.0737391748341673	0.6890390793315960	0.0724930941906601	T	T	T
0.3291488589647339	0.8229609869047638	0.2318336684802915	T	T	T
0.1699848166589434	0.9374365065331929	0.1572621603996476	T	T	T
0.9197999840000008	0.8067899939999990	0.0135999999999967	F	F	F
0.2947399910000001	0.1418000010000000	0.0581299999999985	F	F	F
0.5378213339346609	0.2750034890839627	0.1994485807338517	T	T	T
0.7060099839999978	0.3580600020000019	0.0706700010000034	F	F	F
0.9658056330871342	0.4857131753130977	0.1973348877026325	T	T	T
0.2745615508911609	0.3879125924258842	0.0899383389633357	T	T	T
0.5334454813288527	0.5269771454237050	0.1941342645468733	T	T	T
0.7391800279999998	0.1139999999999972	0.0271799989999977	F	F	F
0.9942675961418723	0.2352458187073081	0.1700730594786513	T	T	T
0.8231881849976173	0.0760159887820385	0.1651202379436259	T	T	T
0.5987344345813215	0.9152156089762797	0.1047568882409281	T	T	T

0.3866899909999972	0.5958499910000015	0.0069799999999987	F	F	F
0.6439275862297958	0.7088837097605706	0.1587087311777466	T	T	T
0.2279904298263047	0.6201530749419980	0.0923249366682642	T	T	T
0.5219930376075459	0.7818727128282218	0.2335804610877047	T	T	T
0.9628285079087866	0.9769093794344527	0.1771759297183783	T	T	T
0.7508699889999946	0.8702700139999991	0.0216899999999995	F	F	F
0.0805003145485386	0.1632913049377621	0.0596631842181953	T	T	T
0.3233038581649454	0.3122771652435931	0.2309307070128185	T	T	T
0.1501236953039940	0.4672587566869798	0.1530694488549776	T	T	T
0.8750200269999991	0.3507800100000011	0.0000000000000000	F	F	F
0.5651468562389393	0.4511031006800171	0.0679101561705508	T	T	T
0.8346732787027319	0.5749682592345019	0.2225105411751039	T	T	T
0.4184600110000005	0.0453499999999991	0.0133399999999995	F	F	F
0.6741855540644672	0.1778085974884752	0.1581706281405782	T	T	T
0.5465916336365775	0.0255783057990704	0.1804137828570646	T	T	T
0.2934199870000000	0.8915699719999993	0.0453899990000011	F	F	F
0.7165201043189005	0.6101142863923451	0.0858348015472713	T	T	T
0.9640434843961698	0.7306151804346344	0.1881385146883367	T	T	T
0.2467399980000044	0.5069199799999993	0.0306100009999994	F	F	F
0.4596650422979841	0.6574965799326802	0.1976498769876137	T	T	T
0.0228406248533863	0.1000659063485290	0.1851979429197871	T	T	T
0.7677420981783244	0.9843320813176649	0.0802357664889969	T	T	T
0.1783312279682442	0.1746996471214066	0.1677961549392759	T	T	T
0.9242016356750892	0.0603608701080074	0.0627928797153435	T	T	T
0.1217800009999978	0.4070599969999975	0.0026000000000010	F	F	F
0.3428373817970516	0.5398993488139875	0.1569854903438941	T	T	T
0.0342997363309724	0.6115196501380233	0.1941923370467234	T	T	T
0.7684297734758534	0.4883410422659953	0.0437662323234957	T	T	T
0.2270399929999991	0.0167599990000014	0.0415600019999971	F	F	F
0.4762011882043454	0.1462644186394414	0.1887233454429160	T	T	T
0.3407645582703110	0.0474085178079914	0.1589272072919740	T	T	T
0.1020800020000010	0.9169099929999973	0.0135599999999982	F	F	F
0.1702280722185947	0.7141505142510914	0.1749097572658761	T	T	T
0.9105713309263285	0.5720939839928352	0.0906621645124261	T	T	T
0.2179286709758503	0.2260907006686368	0.4340183552225065	T	T	T
0.1494684882047181	0.2390320707328147	0.4728312058318391	T	T	T
0.1374280913946307	0.2959734749018074	0.4961251886280322	T	T	T
0.1949366462291366	0.3396740174454146	0.4799564006810896	T	T	T
0.2638862248601594	0.3267364696107865	0.4416547383532988	T	T	T
0.2776794734529610	0.2693457420623421	0.4176573470479253	T	T	T
0.3512401995076541	0.2514346263185746	0.3814333337402163	T	T	T
0.4222102242448251	0.2826549750753652	0.3664599487758248	T	T	T
0.2273340993947483	0.1810996721360808	0.4172733017911907	T	T	T
0.1055694860925251	0.2044183361077593	0.4856697535635056	T	T	T

0.0842796256309120	0.3062061771002256	0.5270467725156498	T	T	T
0.1861492810669214	0.3841349920753947	0.4983371920737110	T	T	T
0.3085332991290052	0.3614125475181368	0.4309673402027859	T	T	T
0.3282166656226009	0.3058886414315341	0.2779356319312321	T	T	T
0.3510166840440347	0.2049142060151502	0.3679063936087451	T	T	T
0.4319238820836682	0.3268230693722085	0.3855131568380205	T	T	T
0.4417185148837386	0.3055902670733958	0.2840852815442779	T	T	T
0.4780483454463090	0.2579458120117725	0.3546884924977460	T	T	T

3. TS-[II’-III’]

```
   1.00000000000000
     16.1327991486000002    0.0000000000000000    0.0000000000000000
     -0.0242462366000000   22.8120865715000001    0.0000000000000000
      0.0000000000000000    0.0000000000000000   20.4906997681000007
   Fe   K   O   C   H
     32   32   64   8   10
Selective dynamics
Direct
```

0.3120000000000008	0.5663300189999987	0.0711500000000029	F	F	F
0.5455380823785629	0.7080180633485734	0.1986998364184660	T	T	T
0.9330472249851977	0.0562907985113162	0.1592351242858621	T	T	T
0.6887500229999972	0.9335399349999989	0.0576500000000024	F	F	F
0.0666065380125576	0.1729772468724842	0.1605251045957718	T	T	T
0.8137500560000127	0.0563699980000010	0.0600599990000035	F	F	F
0.1870000099999984	0.4435000180000017	0.0687399999999982	F	F	F
0.4387353894718501	0.5734168670188873	0.1918475247564538	T	T	T
0.6779199550000001	0.4389499729999997	0.0636799979999978	F	F	F
0.9351682803067504	0.5654185764366696	0.1878660006481827	T	T	T
0.3228299770000002	0.0609200009999995	0.0651199960000000	F	F	F
0.5667185338706147	0.2001356325394411	0.1932042016893435	T	T	T
0.4485374810368721	0.0695583223317486	0.1810928833393446	T	T	T
0.1978400080000000	0.9380800289999982	0.0627100020000029	F	F	F
0.0607889546239184	0.6887195348240051	0.1700284775707935	T	T	T
0.8029099399999993	0.5617799750000003	0.0660900029999993	F	F	F
0.1865600029999982	0.6926499989999968	0.0732799970000002	F	F	F
0.4353408753998763	0.8290095915489224	0.1985923377961030	T	T	T
0.0583130437533312	0.9319916988605684	0.1764211999347106	T	T	T
0.8141899450000025	0.8072100050000017	0.0555200020000015	F	F	F
0.1891900079999977	0.1794600099999997	0.0585899989999987	F	F	F
0.4400006396735144	0.3193150613940032	0.1901121207524417	T	T	T
0.0582648335227559	0.4375533416892980	0.1973073228755166	T	T	T
0.8115599979999999	0.3204100060000030	0.0702100000000030	F	F	F
0.2996900079999991	0.3144599940000035	0.0635899979999976	F	F	F
0.5611835353814366	0.4524906650782376	0.1646575307424885	T	T	T
0.7010599979999981	0.1853999880000003	0.0652100020000006	F	F	F

0.9544809634020349	0.3122138834600727	0.1931627576875436	T	T	T
0.3260699840000001	0.8131599539999996	0.0621399990000029	F	F	F
0.5709409614056099	0.9465526369726879	0.1951249013213713	T	T	T
0.6746799789999969	0.6867099999999979	0.0666599999999988	F	F	F
0.9311680315313042	0.8088570535090260	0.1791378666757218	T	T	T
0.4349099740000053	0.6928799720000001	0.0672200030000027	F	F	F
0.6747718945477840	0.8232311334585125	0.2208839558783822	T	T	T
0.5658399890000027	0.8069900240000011	0.0615799959999990	F	F	F
0.8148851732836200	0.9405742043545541	0.2003536320783274	T	T	T
0.1781077102836935	0.2934777274066659	0.2054275334165474	T	T	T
0.9408499919999995	0.1848999919999983	0.0583400000000012	F	F	F
0.0598999990000024	0.3149699820000009	0.0704599999999971	F	F	F
0.3090481891063618	0.4352388021019190	0.2079533937216697	T	T	T
0.5548899509999983	0.3124599909999972	0.0676700040000000	F	F	F
0.8193570361083962	0.4518235535991495	0.2286176878129680	T	T	T
0.4458600110000006	0.1874099830000020	0.0611300010000022	F	F	F
0.6885195153486338	0.3293493476186706	0.1960793081365304	T	T	T
0.0708500000000001	0.8094900019999970	0.0643699979999965	F	F	F
0.3179272504734710	0.9413063018822257	0.2175549343363807	T	T	T
0.1713914196779587	0.8203805827867272	0.2296229017988245	T	T	T
0.9299000150000012	0.6903700020000016	0.0644299960000012	F	F	F
0.0640299949999988	0.5736399899999967	0.0724299979999969	F	F	F
0.3213982196383454	0.7096297302503489	0.2257353463804801	T	T	T
0.1829461733246000	0.0589182779916909	0.1915277850300866	T	T	T
0.9367200120000021	0.9262200140000019	0.0563700009999977	F	F	F
0.0617299979999974	0.0552100009999990	0.0519199989999990	F	F	F
0.3292804130862247	0.1926811852099673	0.1888180684962888	T	T	T
0.1883345358608995	0.5724256159027569	0.2055283177920100	T	T	T
0.9390199930000022	0.4446499660000001	0.0768800009999993	F	F	F
0.4194500130000035	0.4348600000000004	0.0659700060000006	F	F	F
0.6750257243099916	0.5758276497623734	0.2089109834928084	T	T	T
0.5813000079999995	0.0650099949999969	0.0628299990000016	F	F	F
0.8299840261412765	0.1926292730493051	0.1915727987618145	T	T	T
0.6831087639548759	0.0671429515596744	0.2289713745696171	T	T	T
0.4562999970000024	0.9360200129999967	0.0672700009999971	F	F	F
0.5444600149999989	0.5638499820000007	0.0615299990000011	F	F	F
0.8085429168522701	0.6896248535968338	0.1985835619961435	T	T	T
0.2598246298317200	0.7509761903310155	0.0449600098273827	T	T	T
0.4696397710796280	0.9080810255726935	0.2013225587684170	T	T	T
0.0238407239256463	0.8553759619261511	0.1885341875353916	T	T	T
0.7648800200000053	0.7331200089999967	0.0370399990000010	F	F	F
0.4048226567217700	0.2940393278551856	0.1065612608450370	T	T	T
0.6751667473554100	0.4453088660362436	0.1675926070941775	T	T	T
0.6109799700000025	0.2119999880000023	0.0135300000000029	F	F	F

0.8491062142095025	0.3332894613749291	0.1603330110918272	T	T	T
0.2089899830000034	0.2610599960000002	0.0681299999999965	F	F	F
0.4763853509446586	0.3964921560600216	0.1830166889439012	T	T	T
0.0445204200184729	0.3595777215692202	0.2198944527498340	T	T	T
0.7917600230000053	0.2388100000000009	0.0606699989999981	F	F	F
0.4246662178455276	0.8068745693284917	0.1041934215520884	T	T	T
0.6642325927700349	0.9409872945698594	0.2398164155395369	T	T	T
0.5840700500000011	0.6907699570000005	0.0071999999999974	F	F	F
0.8224579138988054	0.8206837959348600	0.1507307555960902	T	T	T
0.0738406682916625	0.6878057926652332	0.0723176199478552	T	T	T
0.3310472254494614	0.8231924656359739	0.2289610522002370	T	T	T
0.1704280949494357	0.9374948023677410	0.1573740354055785	T	T	T
0.9197999660000011	0.8067899420000018	0.0136000010000004	F	F	F
0.2947399959999970	0.1417999989999998	0.0581299970000018	F	F	F
0.5314838958416026	0.2764688194634194	0.2274822196372648	T	T	T
0.7060100099999996	0.3580599940000013	0.0706700020000000	F	F	F
0.9654266392549230	0.4859925157588710	0.1984519221974923	T	T	T
0.2708192799952743	0.3905931078272151	0.0933978937379407	T	T	T
0.5337859846816169	0.5265423091818218	0.1946839415333256	T	T	T
0.7391800589999988	0.1139999979999970	0.0271800000000013	F	F	F
0.9909521700917746	0.2348192405126096	0.1712811801705197	T	T	T
0.8221686969192994	0.0744206439026132	0.1652943832210677	T	T	T
0.5992223854395222	0.9162913383823635	0.1054867198791040	T	T	T
0.3866899799999999	0.5958499979999985	0.0069799999999987	F	F	F
0.6476917122144884	0.7086314573635971	0.1556752749294385	T	T	T
0.2286861154907827	0.6203230341496250	0.0927267603774829	T	T	T
0.5226321051643861	0.7822936945066552	0.2295809734137382	T	T	T
0.9642947047406434	0.9783761001057487	0.1737732727975874	T	T	T
0.7508699959999987	0.8702699959999974	0.0216899999999995	F	F	F
0.0809810694669554	0.1628167763950311	0.0631795507997142	T	T	T
0.3404800547791151	0.3114762875360877	0.2442028327346870	T	T	T
0.1452979010394705	0.4706901680638286	0.1502208429855795	T	T	T
0.8750200690000014	0.3507799989999967	0.0000000000000000	F	F	F
0.5656046217389867	0.4523052680864373	0.0672587421572035	T	T	T
0.8346555408434536	0.5755012964523872	0.2229661782192134	T	T	T
0.4184600070000002	0.0453499999999991	0.0133400010000031	F	F	F
0.6720346802609933	0.1842055266440694	0.1585303679044615	T	T	T
0.5461306709711637	0.0257264648828345	0.1832651519665988	T	T	T
0.2934199609999979	0.8915699790000033	0.0453899990000011	F	F	F
0.7156045858589637	0.6093214759456846	0.0841982735246514	T	T	T
0.9642449519384706	0.7313459494859362	0.1873943308829903	T	T	T
0.2467399929999985	0.5069199550000008	0.0306100009999994	F	F	F
0.4601847845688840	0.6534360935805519	0.2001829413630224	T	T	T
0.0224159582618495	0.1009903920135974	0.1871704381713228	T	T	T

0.7698011879898414	0.9836544797030197	0.0777301161301956	T	T	T
0.1778448259730792	0.1765824799795565	0.1739516167929788	T	T	T
0.9238745395670309	0.0651296783811518	0.0623871668771710	T	T	T
0.1217800000000011	0.4070599889999968	0.0026000000000010	F	F	F
0.3432742545027559	0.5394450308433411	0.1567294611776198	T	T	T
0.0339456330144268	0.6119691052056127	0.1944171464847984	T	T	T
0.7699664233014968	0.4874834379907424	0.0452223003536799	T	T	T
0.2270399949999984	0.0167599990000014	0.0415600010000006	F	F	F
0.4769589962678719	0.1487142543843523	0.1845063415946731	T	T	T
0.3411419545615392	0.0469040683422844	0.1584179509264874	T	T	T
0.1020800040000022	0.9169099779999996	0.0135599990000017	F	F	F
0.1696595119370308	0.7150346150077093	0.1743481808509655	T	T	T
0.9102361572156894	0.5723192458311371	0.0912449460491495	T	T	T
0.3222059097278175	0.2213662344980827	0.4140189485714179	T	T	T
0.2452619463426277	0.2328959898331064	0.4413857715828858	T	T	T
0.2272613194767480	0.2890183292104399	0.4654848653219754	T	T	T
0.2875264767123831	0.3332609239218406	0.4616690773763463	T	T	T
0.3645395688588854	0.3223524020726416	0.4336332103690984	T	T	T
0.3842949759946529	0.2656441881334578	0.4088404129109916	T	T	T
0.4627304791404864	0.2502005291354457	0.3814522876403240	T	T	T
0.5280874524658724	0.2871340747379775	0.3586639565755951	T	T	T
0.3366664691930991	0.1774553770157571	0.3960335212783675	T	T	T
0.1990610547068945	0.1980505084327790	0.4446295201046618	T	T	T
0.1672765866053456	0.2979092987854313	0.4879525893732281	T	T	T
0.2741910792194229	0.3764969140123254	0.4818765126588240	T	T	T
0.4112054800676896	0.3570008480365867	0.4326120770165442	T	T	T
0.3529839246405027	0.3078145503862487	0.2905700165024992	T	T	T
0.4711400696783057	0.2031661379665127	0.3728121554864723	T	T	T
0.5250849991306005	0.3336185182579922	0.3721974322188217	T	T	T
0.5272174291566947	0.2834749374145665	0.2964791305101445	T	T	T
0.5904042156105918	0.2687919817163766	0.3665689120540910	T	T	T

4. TS-[IV-V]

1.00000000000000

16.1327991486000002	0.0000000000000000	0.0000000000000000
-0.0242462366000000	22.8120865715000001	0.0000000000000000
0.0000000000000000	0.0000000000000000	20.4906997681000007

Fe	K	O	H
32	32	64	2

Selective dynamics

Direct

0.3120000059999981	0.5663300160000020	0.0711499969999991	F	F	F
0.5443968049172696	0.7084051708859462	0.1984497713912532	T	T	T
0.9331309729507780	0.0552834255412620	0.1602367596609469	T	T	T
0.6887500289999977	0.9335399870000032	0.0576500000000024	F	F	F

0.0669134754302334	0.1719616240069044	0.1567267769615555	T	T	T
0.8137500289999977	0.0563700009999977	0.0600599979999998	F	F	F
0.1870000059999981	0.4435000120000012	0.0687400030000020	F	F	F
0.4377520160928299	0.5748041988319841	0.1913071606345937	T	T	T
0.6779199839999990	0.4389500019999986	0.0636800010000016	F	F	F
0.9352223279981899	0.5649547032455047	0.1875350917473000	T	T	T
0.3228299920000097	0.0609200000000030	0.0651199969999965	F	F	F
0.5705126122821085	0.1959537255582475	0.1856516713419083	T	T	T
0.4487013533326021	0.0684020484492223	0.1810460494979793	T	T	T
0.1978400050000033	0.9380800129999969	0.0627100020000029	F	F	F
0.0610360203237121	0.6885019536171626	0.1705797563797091	T	T	T
0.8029099699999946	0.5617799759999969	0.0660900029999993	F	F	F
0.1865600049999969	0.6926500200000021	0.0732799990000004	F	F	F
0.4350724789615654	0.8289463592349406	0.1972223884685604	T	T	T
0.0574697109757096	0.9315542100504501	0.1774286688022357	T	T	T
0.8141899700000010	0.8072100280000001	0.0555200020000015	F	F	F
0.1891900000000035	0.1794600039999992	0.0585899989999987	F	F	F
0.4497878105161774	0.3198989228411406	0.1901274970977482	T	T	T
0.0593774688415394	0.4371901726923326	0.1982814515737772	T	T	T
0.8115599750000015	0.3204100130000000	0.0702100020000032	F	F	F
0.2996900080000060	0.3144600090000012	0.0635899979999976	F	F	F
0.5608562022449206	0.4533792002981158	0.1650045805710860	T	T	T
0.7010599970000015	0.1853999940000008	0.0652100000000004	F	F	F
0.9549708685710598	0.3127174605528822	0.1926007747784553	T	T	T
0.3260700109999988	0.8131600020000036	0.0621399990000029	F	F	F
0.5704290955673557	0.9458909655383039	0.1945296981869618	T	T	T
0.6746799949999982	0.6867099999999979	0.0666600019999990	F	F	F
0.9312572157553376	0.8081694588792629	0.1791607715109222	T	T	T
0.4349099989999985	0.6928799749999968	0.0672200019999991	F	F	F
0.6741971241670726	0.8225756831396112	0.2219835282538298	T	T	T
0.5658400060000019	0.8069900269999977	0.0615799990000028	F	F	F
0.8134875507839752	0.9397224740605949	0.2007009643667317	T	T	T
0.1696562325803868	0.2891651711747372	0.2084682315122421	T	T	T
0.9408500190000098	0.1849000010000026	0.0583400020000013	F	F	F
0.0599000010000026	0.3149699869999978	0.0704599990000006	F	F	F
0.3092895585933491	0.4353601747190083	0.2078244052358045	T	T	T
0.5548899770000020	0.3124600049999984	0.0676700030000035	F	F	F
0.8202416267652666	0.4521920484917956	0.2288957911735778	T	T	T
0.4458599980000031	0.1874099970000032	0.0611299979999984	F	F	F
0.6904349475295888	0.3315034632910496	0.1975250052324284	T	T	T
0.0708500000000001	0.8094900250000023	0.0643699990000002	F	F	F
0.3171826849057169	0.9412812661260475	0.2175400063226078	T	T	T
0.1710124219773377	0.8200817269920506	0.2279532974759420	T	T	T
0.9298999909999992	0.6903700229999998	0.0644299980000014	F	F	F

0.0640299989999988	0.5736399890000001	0.0724299999999971	F	F	F
0.3205937740990734	0.7104312931069003	0.2295784712639417	T	T	T
0.1826106334351181	0.0576857092978500	0.1908780139287103	T	T	T
0.9367200140000023	0.9262200000000007	0.0563700009999977	F	F	F
0.0617300010000008	0.0552100020000026	0.0519200000000026	F	F	F
0.3302648404271480	0.1904929974125551	0.1895645378426281	T	T	T
0.1885588193679766	0.5720758423053383	0.2052226540408668	T	T	T
0.9390199780000046	0.4446499940000024	0.0768800009999993	F	F	F
0.4194500150000025	0.4348599910000033	0.0659700040000004	F	F	F
0.6751556474577072	0.5750807375910298	0.2099116109490851	T	T	T
0.5813000200000005	0.0650099960000006	0.0628300010000018	F	F	F
0.8309474936554366	0.1920907296285037	0.1917701922508570	T	T	T
0.6833690794112002	0.0664114376563063	0.2280788606324791	T	T	T
0.4562999899999980	0.9360200169999970	0.0672700029999973	F	F	F
0.5444599989999972	0.5638499860000010	0.0615300010000013	F	F	F
0.8077668009418275	0.6892978694452268	0.1981479883038588	T	T	T
0.2586381498313486	0.7515397255205869	0.0447996096492886	T	T	T
0.4685020392758190	0.9081531600439092	0.2006226660290835	T	T	T
0.0238435472436365	0.8545071346353765	0.1882965237950103	T	T	T
0.7648800020000195	0.7331200240000015	0.0370399990000010	F	F	F
0.3964857888374667	0.2930810740080148	0.1050048672025028	T	T	T
0.6749535494654495	0.4467652147830350	0.1676637018042229	T	T	T
0.6109799740000028	0.2119999969999995	0.0135300000000029	F	F	F
0.8493398939415755	0.3332705120348507	0.1602936973107560	T	T	T
0.2089899930000030	0.2610599989999969	0.0681300010000001	F	F	F
0.4737694198004831	0.3990393031687915	0.1852192279751819	T	T	T
0.0453405629191366	0.3587600520641416	0.2212572796529457	T	T	T
0.7917600270000023	0.2388100029999976	0.0606700000000018	F	F	F
0.4251172314666007	0.8064603981155969	0.1030815022796574	T	T	T
0.6625866874932740	0.9396677960070150	0.2404117755290400	T	T	T
0.5840700270000028	0.6907699699999981	0.0071999999999974	F	F	F
0.8227840888206046	0.8197472281245268	0.1508275147325477	T	T	T
0.0737034867480801	0.6887763929158410	0.0729577495214428	T	T	T
0.3296983679446939	0.8235847714435920	0.2254282066001636	T	T	T
0.1693211680909741	0.9379504178479579	0.1573075939180697	T	T	T
0.9197999840000008	0.8067899939999990	0.0135999999999967	F	F	F
0.2947399910000001	0.1418000010000000	0.0581299999999985	F	F	F
0.5452458883030218	0.2771207424166633	0.1964978577821483	T	T	T
0.7060099839999978	0.3580600020000020	0.0706700010000034	F	F	F
0.9658436276908802	0.4857623063707237	0.1974881611008996	T	T	T
0.2746557366817299	0.3869032678964643	0.0925173359678837	T	T	T
0.5331510999329296	0.5278884088515809	0.1928441324763384	T	T	T
0.7391800279999998	0.1139999999999972	0.0271799989999977	F	F	F
0.9966740417558526	0.2364095425412742	0.1685355967666240	T	T	T

0.8231068237484820	0.0750116680248354	0.1657417515832904	T	T	T
0.5990250163643580	0.9156877885144397	0.1050253263003071	T	T	T
0.3866899909999972	0.5958499910000015	0.0069799999999987	F	F	F
0.6462809499303503	0.7098869901939153	0.1552575689475191	T	T	T
0.2283137913479294	0.6201336702944573	0.0922056369051560	T	T	T
0.5217565474297672	0.7825231689106005	0.2301728616623464	T	T	T
0.9627265304234811	0.9773104087146578	0.1768474631156991	T	T	T
0.7508699889999946	0.8702700139999990	0.0216899999999995	F	F	F
0.0806722452332238	0.1631652125057954	0.0586991603859133	T	T	T
0.3328537845181572	0.3065839437118072	0.2479926007224762	T	T	T
0.1512498362273931	0.4674108908959980	0.1529756206055097	T	T	T
0.8750200269999979	0.3507800100000011	0.0000000000000000	F	F	F
0.5649855058105189	0.4511314410383847	0.0678596970102845	T	T	T
0.8343734938533061	0.5749840465887459	0.2222459004401287	T	T	T
0.4184600110000005	0.0453499999999991	0.0133399999999995	F	F	F
0.6766841659148852	0.1751045650392911	0.1577275367317625	T	T	T
0.5459901736418544	0.0249324735857357	0.1810197380548004	T	T	T
0.2934199870000000	0.8915699719999992	0.0453899990000011	F	F	F
0.7162470164720105	0.6098404009975127	0.0853783704041575	T	T	T
0.9640698425032923	0.7308226469072068	0.1880457482313886	T	T	T
0.2467399980000025	0.5069199799999993	0.0306100009999994	F	F	F
0.4572947016671094	0.6551844672797600	0.2003224753954811	T	T	T
0.0232326538253176	0.1011117127333137	0.1862469371057216	T	T	T
0.7687439613659208	0.9839889136044491	0.0793940192640861	T	T	T
0.1795829504405972	0.1749735456788883	0.1666745734665369	T	T	T
0.9241748314851534	0.0624733600053418	0.0635927783018104	T	T	T
0.1217800009999976	0.4070599969999975	0.0026000000000010	F	F	F
0.3431696835365869	0.5392274167914266	0.1565502682731221	T	T	T
0.0340542081161956	0.6116651432935498	0.1942801544869752	T	T	T
0.7681097789687215	0.4883158412710387	0.0435899757288141	T	T	T
0.2270399929999982	0.0167599990000014	0.0415600019999971	F	F	F
0.4774823988770649	0.1470362797530527	0.1875860744950018	T	T	T
0.3408748738371230	0.0475645153123097	0.1589209204499937	T	T	T
0.1020800020000010	0.9169099929999973	0.0135599999999982	F	F	F
0.1705290808006978	0.7136190559851414	0.1752558998300628	T	T	T
0.9105804613158170	0.5718933467569940	0.0904861378927393	T	T	T
0.3313307249609210	0.3074038897070679	0.2956048232769533	T	T	T
0.4305169087677712	0.3135876032198368	0.2632302736146386	T	T	T

5. TS-[IV'-V']

1.00000000000000

16.1327991486000002	0.0000000000000000	0.0000000000000000
-0.0242462366000000	22.8120865715000001	0.0000000000000000
0.0000000000000000	0.0000000000000000	20.4906997681000007

Fe K O H

32 32 65 4

Selective dynamics

Direct

0.3120000000000008	0.5663300189999987	0.0711500000000029	F	F	F
0.5450047001717510	0.7082496408061325	0.1984435921917955	T	T	T
0.9337024159722364	0.0564042113064401	0.1593243301458411	T	T	T
0.6887500229999972	0.9335399349999989	0.0576500000000024	F	F	F
0.0676013304193366	0.1726002532696721	0.1580865889652172	T	T	T
0.8137500560000127	0.0563699980000010	0.0600599990000035	F	F	F
0.1870000099999984	0.4435000180000017	0.0687399999999982	F	F	F
0.4380552612501359	0.5750553322481022	0.1910182181963813	T	T	T
0.6779199550000001	0.4389499729999997	0.0636799979999978	F	F	F
0.9354618284456503	0.5653142592147887	0.1881468663358135	T	T	T
0.3228299770000002	0.0609200009999995	0.0651199960000000	F	F	F
0.5691380809552988	0.1964736675660840	0.1910122292688258	T	T	T
0.4486884123880551	0.0687062819584894	0.1801744127437607	T	T	T
0.1978400080000000	0.9380800289999982	0.0627100020000029	F	F	F
0.0607719468694541	0.6887593654663340	0.1701289346247885	T	T	T
0.8029099399999993	0.5617799750000003	0.0660900029999993	F	F	F
0.1865600029999982	0.6926499989999968	0.0732799970000002	F	F	F
0.4353525690679507	0.8291010276580402	0.1973843153754539	T	T	T
0.0586623397622458	0.9322976838019946	0.1763704415140622	T	T	T
0.8141899450000025	0.8072100050000017	0.0555200020000015	F	F	F
0.1891900079999977	0.1794600099999997	0.0585899989999987	F	F	F
0.4528514958128009	0.3236865482952591	0.1822314513129314	T	T	T
0.0590996146918896	0.4371322594322948	0.1981371706925854	T	T	T
0.8115599979999999	0.3204100060000030	0.0702100000000030	F	F	F
0.2996900079999991	0.3144599940000035	0.0635899979999976	F	F	F
0.5604923015675040	0.4552215205700036	0.1652058362462890	T	T	T
0.7010599979999981	0.1853999880000003	0.0652100020000006	F	F	F
0.9544681311587865	0.3124438592904176	0.1929938873293008	T	T	T
0.3260699840000001	0.8131599539999996	0.0621399990000029	F	F	F
0.5710993376714536	0.9464639512324015	0.1951327160258030	T	T	T
0.6746799789999969	0.6867099999999979	0.0666599999999988	F	F	F
0.9314614882783033	0.8090743490406941	0.1788123739709720	T	T	T
0.4349099740000120	0.6928799720000001	0.0672200030000027	F	F	F
0.6748106722280508	0.8231260020122201	0.2209979366346917	T	T	T
0.5658399890000041	0.8069900240000011	0.0615799959999990	F	F	F
0.8148841515344273	0.9410662529295587	0.2006077062922677	T	T	T
0.1719395340867782	0.2910835174389501	0.2070487797636440	T	T	T
0.9408499919999995	0.1848999919999983	0.0583400000000012	F	F	F
0.0598999990000024	0.3149699820000009	0.0704599999999971	F	F	F
0.3104908993479720	0.4367936917187109	0.2104175300039007	T	T	T
0.5548899509999983	0.3124599909999972	0.0676700040000000	F	F	F

0.8195952685671084	0.4526222431338480	0.2284141688927115	T	T	T
0.4458600110000006	0.1874099830000020	0.0611300010000022	F	F	F
0.6894367175757884	0.3302611427587372	0.1971213061285215	T	T	T
0.0708500000000001	0.8094900019999970	0.0643699979999965	F	F	F
0.3181898070732822	0.9413698037719388	0.2184887257289820	T	T	T
0.1713454108878885	0.8210488844650849	0.2281636663097678	T	T	T
0.9299000150000012	0.6903700020000016	0.0644299960000012	F	F	F
0.0640299949999988	0.5736399899999967	0.0724299979999969	F	F	F
0.3200270640586964	0.7103311429988197	0.2276113868633848	T	T	T
0.1832672331460384	0.0579600195763224	0.1900292392964363	T	T	T
0.9367200120000021	0.9262200140000019	0.0563700009999977	F	F	F
0.0617299979999974	0.0552100009999990	0.0519199989999990	F	F	F
0.3301107800263728	0.1897119127034971	0.2021953175015994	T	T	T
0.1882144287963383	0.5717194822656804	0.2054927623008153	T	T	T
0.9390199930000022	0.4446499660000001	0.0768800009999993	F	F	F
0.4194500130000035	0.4348600000000004	0.0659700060000006	F	F	F
0.6767582859513989	0.5769188201580440	0.2096612236451041	T	T	T
0.5813000079999995	0.0650099949999969	0.0628299990000016	F	F	F
0.8305749587740628	0.1929884467294173	0.1917885979229981	T	T	T
0.6837077444505389	0.0672803223927087	0.2293282254217947	T	T	T
0.4562999970000024	0.9360200129999967	0.0672700009999971	F	F	F
0.5444600149999989	0.5638499820000007	0.0615299990000011	F	F	F
0.8089992101953556	0.6898273551192344	0.1987026790028266	T	T	T
0.2598411568601176	0.7509282377385854	0.0449570219280915	T	T	T
0.4697108338437449	0.9081493360289709	0.2011984509714030	T	T	T
0.0238728167991117	0.8555551078333360	0.1882583615326338	T	T	T
0.7648800200000068	0.7331200089999967	0.0370399990000010	F	F	F
0.3980307462037427	0.2927812305349447	0.1036919880499328	T	T	T
0.6743552709895866	0.4461333715800290	0.1677562242963839	T	T	T
0.6109799700000025	0.2119999880000023	0.0135300000000029	F	F	F
0.8489157856493292	0.3330635715402023	0.1603317704476268	T	T	T
0.2089899830000034	0.2610599960000002	0.0681299999999965	F	F	F
0.4713687031638056	0.4043947132987555	0.1859713908854967	T	T	T
0.0448374430446984	0.3587061579729963	0.2209490785469073	T	T	T
0.7917600230000053	0.2388100000000009	0.0606699989999981	F	F	F
0.4252423160575238	0.8070803393002262	0.1031969830634641	T	T	T
0.6641492176515579	0.9408414142562128	0.2400410615858139	T	T	T
0.5840700500000011	0.6907699570000005	0.0071999999999974	F	F	F
0.8226575405416318	0.8203156177496117	0.1508734577257798	T	T	T
0.0738459967785257	0.6878201189852252	0.0724352841017039	T	T	T
0.3305153112253504	0.8235210709904591	0.2268441470979905	T	T	T
0.1709187551611835	0.9377083591650163	0.1573853018085194	T	T	T
0.9197999660000011	0.8067899420000018	0.0136000010000004	F	F	F
0.2947399959999970	0.1417999989999998	0.0581299970000018	F	F	F

0.5376691755878549	0.2740323726554422	0.2233543785814903	T	T	T
0.7060100099999996	0.3580599940000013	0.0706700020000000	F	F	F
0.9658171334439392	0.4859116025798140	0.1973071733847039	T	T	T
0.2740486961356848	0.3859494656657597	0.0946589533668619	T	T	T
0.5355993278775291	0.5309705134690943	0.1920360977531513	T	T	T
0.7391800589999988	0.1139999979999970	0.0271800000000013	F	F	F
0.9946604196760921	0.2356343076130495	0.1700662500040959	T	T	T
0.8231695118083708	0.0749828147602685	0.1657226106075025	T	T	T
0.5992535861965622	0.9161896098138821	0.1054295381046395	T	T	T
0.3866899799999999	0.5958499979999985	0.0069799999999987	F	F	F
0.6472539593870267	0.7090953434828745	0.1556697779526446	T	T	T
0.2284189045614028	0.6201933564109158	0.0927526733015532	T	T	T
0.5223213729520269	0.7824478041737020	0.2294868754215051	T	T	T
0.9642682402207451	0.9784264424639811	0.1737788190169386	T	T	T
0.7508699959999987	0.8702699959999974	0.0216899999999995	F	F	F
0.0806902931615810	0.1636208251763848	0.0601923394919308	T	T	T
0.3062197697515765	0.2928503758731528	0.2878628110925613	T	T	T
0.1511819399913114	0.4673380140843915	0.1530374634198751	T	T	T
0.8750200690000014	0.3507799989999967	0.0000000000000000	F	F	F
0.5653150895478658	0.4515240196605673	0.0673638563686457	T	T	T
0.8351594203285171	0.5759794872058662	0.2236820601089008	T	T	T
0.4184600070000002	0.0453499999999991	0.0133400010000031	F	F	F
0.6747056756697207	0.1801668368467706	0.1580667204235795	T	T	T
0.5466584948510517	0.0255967641658372	0.1828860404160968	T	T	T
0.2934199609999979	0.8915699790000033	0.0453899990000011	F	F	F
0.7159448288059564	0.6095919782685076	0.0844057985068297	T	T	T
0.9641890669926731	0.7315714497202788	0.1872401624828189	T	T	T
0.2467399929999985	0.5069199550000008	0.0306100009999994	F	F	F
0.4579984874757603	0.6550881994706053	0.2008809192498684	T	T	T
0.0249108448053501	0.1007640063143962	0.1858874192568744	T	T	T
0.7698295951144354	0.9835967575087079	0.0784320821396028	T	T	T
0.1803462051323265	0.1761939916799882	0.1676124156311716	T	T	T
0.9239148403122023	0.0651138413961173	0.0623942036221932	T	T	T
0.1217800000000011	0.4070599889999968	0.0026000000000010	F	F	F
0.3435487732373921	0.5393431648216340	0.1567176128297974	T	T	T
0.0344041207217048	0.6119854471324293	0.1944422137274870	T	T	T
0.7698728158526138	0.4874776248252410	0.0452637042849220	T	T	T
0.2270399949999984	0.0167599990000014	0.0415600010000006	F	F	F
0.4762157602427342	0.1478939180321050	0.1828038315804399	T	T	T
0.3408077376527949	0.0469508791503396	0.1585026225126802	T	T	T
0.1020800040000022	0.9169099779999996	0.0135599990000017	F	F	F
0.1694062819870704	0.7152618891576259	0.1743806514361645	T	T	T
0.9101226101150671	0.5723750862293459	0.0912290974427259	T	T	T
0.4524799925330005	0.2812849336475817	0.3218242015298380	T	T	T

0.2756749928336020	0.2906994975886577	0.3287782023876811	T	T	T
0.5046590124073989	0.2752724790252793	0.2726822646160216	T	T	T
0.4713297420106063	0.3120544086354369	0.3510581516410473	T	T	T
0.3714187204444152	0.2894606700309141	0.3044072185380485	T	T	T

Supplementary References

(1) Khanna, L.; Verma, N. K. Synthesis, Characterization and Biocompatibility of Potassium Ferrite Nanoparticles. *J. Mater. Sci. Technol.* **2014**, *30*, 30–36.

(2) Grosvenor, A. P.; Kobe, B. A.; Biesinger, M. C.; McIntyre, N. S. Investigation of Multiplet Splitting of Fe 2p XPS Spectra and Bonding in Iron Compounds. *Surf. Interface Anal.* **2004**, *36* (12), 1564–1574. https://doi.org/10.1002/sia.1984.

(3) Ilton, E. S.; Post, J. E.; Heaney, P. J.; Ling, F. T.; Kerisit, S. N. XPS Determination of Mn Oxidation States in Mn (Hydr)Oxides. *Appl. Surf. Sci.* **2016**, *366*, 475–485. https://doi.org/10.1016/j.apsusc.2015.12.159.

(4) Biesinger, M. C.; Payne, B. P.; Grosvenor, A. P.; Lau, L. W.; Gerson, A. R.; Smart, R. S. C. Resolving Surface Chemical States in XPS Analysis of First Row Transition Metals, Oxides and Hydroxides: Cr, Mn, Fe, Co and Ni. *Appl. Surf. Sci.* **2011**, *257* (7), 2717–2730.

(5) Stoch, J.; Gablankowska-Kukucz, J. The Effect of Carbonate Contaminations on the XPS O 1s Band Structure in Metal Oxides. *Surf. Interface Anal.* **1991**, *17* (3), 165–167. https://doi.org/10.1002/sia.740170308.

(6) Sheel, J. G. P.; Crowe, C. M. Simulation and Optimization of an Existing Ethylbenzene Dehydrogenation Reactor. *Can. J. Chem. Eng.* **1969**, *47* (2), 183–187. https://doi.org/10.1002/cjce.5450470215.

(7) James, D. H.; Castor, W. M. Styrene. In *Ullmann's Encyclopedia of Industrial Chemistry*; American Cancer Society, 2000. https://doi.org/10.1002/14356007.a25_329.

(8) Vasudevan, S.; Rangaiah, G. P.; Konda, N. V. S. N. M.; Tay, W. H. Application and Evaluation of Three Methodologies for Plantwide Control of the Styrene Monomer Plant. *Ind. Eng. Chem. Res.* **2009**, *48* (24), 10941–10961. https://doi.org/10.1021/ie900022h.

(9) Luyben, W. L. Design and Control of the Styrene Process. *Ind. Eng. Chem. Res.* **2011**, *50* (3), 1231–1246. https://doi.org/10.1021/ie100023s.

(10) Dimian, A. C.; Bildea, C. S. Energy Efficient Styrene Process: Design and Plantwide Control. *Ind. Eng. Chem. Res.* **2019**, *58* (12), 4890–4905. https://doi.org/10.1021/acs.iecr.8b05560.
